# Suboxic DOM is bioavailable to surface prokaryotes in a simulated overturn of an oxygen minimum zone, Devil’s Hole, Bermuda

**DOI:** 10.3389/fmicb.2023.1287477

**Published:** 2023-12-20

**Authors:** Rachel J. Parsons, Shuting Liu, Krista Longnecker, Kevin Yongblah, Carys Johnson, Luis M. Bolaños, Jacqueline Comstock, Keri Opalk, Melissa C. Kido Soule, Rebecca Garley, Craig A. Carlson, Ben Temperton, Nicholas R. Bates

**Affiliations:** ^1^Microbial Ecology Laboratory, Bermuda Institute of Ocean Sciences, St. George’s, Bermuda; ^2^Julie Ann Wrigley Global Futures Laboratory, School of Ocean Futures, Arizona State University, Tempe, AZ, United States; ^3^Department of Ecology, Evolution and Marine Biology, Marine Science Institute, University of California, Santa Barbara, California, CA, United States; ^4^Department of Environmental and Sustainability Sciences, Kean University, Union, NJ, United States; ^5^Department of Marine Chemistry and Geochemistry, Woods Hole Oceanographic Institution, Woods Hole, MA, United States; ^6^Department of Biology, University of Syracuse, Syracuse, NY, United States; ^7^School of Biosciences, University of Exeter, Exeter, United Kingdom

**Keywords:** ammonia oxidation, Thaumarcheota, SAR202 clade, carbon fixation, chemoautotrophy, total dissolved amino acids, degradation index

## Abstract

Oxygen minimum zones (OMZs) are expanding due to increased sea surface temperatures, subsequent increased oxygen demand through respiration, reduced oxygen solubility, and thermal stratification driven in part by anthropogenic climate change. Devil’s Hole, Bermuda is a model ecosystem to study OMZ microbial biogeochemistry because the formation and subsequent overturn of the suboxic zone occur annually. During thermally driven stratification, suboxic conditions develop, with organic matter and nutrients accumulating at depth. In this study, the bioavailability of the accumulated dissolved organic carbon (DOC) and the microbial community response to reoxygenation of suboxic waters was assessed using a simulated overturn experiment. The surface inoculated prokaryotic community responded to the deep (formerly suboxic) 0.2 μm filtrate with cell densities increasing 2.5-fold over 6 days while removing 5 μmol L^−1^ of DOC. After 12 days, the surface community began to shift, and DOC quality became less diagenetically altered along with an increase in SAR202, a Chloroflexi that can degrade recalcitrant dissolved organic matter (DOM). Labile DOC production after 12 days coincided with an increase of *Nitrosopumilales,* a chemoautotrophic ammonia oxidizing archaea (AOA) that converts ammonia to nitrite based on the ammonia monooxygenase (*amoA*) gene copy number and nutrient data. In comparison, the inoculation of the deep anaerobic prokaryotic community into surface 0.2 μm filtrate demonstrated a die-off of 25.5% of the initial inoculum community followed by a 1.5-fold increase in cell densities over 6 days. Within 2 days, the prokaryotic community shifted from a *Chlorobiales* dominated assemblage to a surface-like heterotrophic community devoid of *Chlorobiales*. The DOM quality changed to less diagenetically altered material and coincided with an increase in the ribulose-1,5-bisphosphate carboxylase/oxygenase form I (*cbbL*) gene number followed by an influx of labile DOM. Upon reoxygenation, the deep DOM that accumulated under suboxic conditions is bioavailable to surface prokaryotes that utilize the accumulated DOC initially before switching to a community that can both produce labile DOM via chemoautotrophy and degrade the more recalcitrant DOM.

## Introduction

The decline in global oceanic oxygen content has resulted in a four-fold expansion of the oxygen minimum zones (OMZs) (Diaz and Rosenberg, 2008; Stramma et al., 2010; Schmidtko et al., 2017; Stramma and Schmidtko, 2019). Ocean warming and increased upper ocean stratification caused by global climate change will likely lead to ocean deoxygenation with implications for ocean productivity, nutrient cycling, carbon cycling, and marine habitats (Keeling et al., 2010; Stramma and Schmidtko, 2019). In coastal and enclosed seas, oxygen deficiency is often related to eutrophication and high degradation rates of organic matter (Zhang et al., 2010; Maßmig et al., 2019), suggesting that respiration is the main driver of coastal deoxygenation (Diaz et al., 2019; Lønborg et al., 2020). These coastal, semi-enclosed, and enclosed basins such as the Baltic Sea (Berg et al., 2014; Maßmig et al., 2019), Black Sea (Jessen et al., 2017; Suominen et al., 2021), Cariaco Basin (Taylor et al., 2001; Lorenzoni et al., 2013; Rodriguez-Mora et al., 2013), the Chilean Coast (Canfield et al., 2010), and Saanich Inlet (Zaikova et al., 2010) are useful model ecosystems for exploring how ocean deoxygenation influences microbial community responses and organic carbon turnover rates (Wright et al., 2012). However, many of these model OMZs are either permanently stratified or undergo mixing events that are unpredictable.

Devil’s Hole, Bermuda is a seasonal OMZ, with reliable transitions and a suboxic layer that occurs within the euphotic zone, making it different from other OMZs (Parsons et al., 2015). As a result, the Devil’s Hole OMZ is a model coastal ecosystem for the study of microbial processes and community succession associated with the seasonal development of hypoxia and anoxia in its deep waters (Thorstenson and Mackenzie, 1974; Andersson et al., 2007; Parsons et al., 2015; Bates, 2017). Devil’s Hole, a collapsed cave in Harrington Sound, Bermuda, has a depth of 25 m where light remains above the 1% light level to the bottom (Manuel et al., 2013; Parsons et al., 2015). During thermally driven stratification, suboxic conditions develop in the summer months below the base of the seasonal thermocline (>20 m) followed by ventilation and reoxygenation during cooler and stormier periods in late Fall. As oxygen levels are depleted to <20 μmol L^−1^ below 22 m, the bacterioplankton community becomes dominated by the low light-adapted photolithoautotrophic H_2_S oxidizer *Chlorobiales* (Kim and Chang, 1991; Overmann et al., 1992; Parsons et al., 2015). This lineage is capable of fixing CO_2_ to organic matter under suboxic or anoxic water conditions (Taipale et al., 2009). Members of the *Thaumarchaeota* and *Euryarcheota* clades are also present in elevated abundances in the suboxic bottom waters (Parsons et al., 2015). Following convective mixing, the prokaryotic community of the entire oxygenated water column is dominated by members of *Synechococcus* and SAR11 (Parsons et al., 2015).

Taxonomic surveys of small-subunit ribosomal RNA (SSU rRNA) gene sequences show that there are similar microbial communities when comparing open ocean OMZs to coastal OMZs (Wright et al., 2012). Dominant bacterial groups within OMZs include chemoorganoheterotrophs such as SAR11, SAR324 and *Roseobacter* that have been shown to assimilate inorganic carbon (Thrash et al., 2010; Swan et al., 2011; Tang et al., 2016; Jing et al., 2022). Other members such as the *Roseobacte*r clade and the Chloroflexi, SAR202 involved with mixotrophic carbon metabolism, methanogenesis and aromatic compound degradation (Moran and Miller, 2007; Swan et al., 2011; Sheik et al., 2014; Cao et al., 2016; Boeuf et al., 2021). In the Bohai Sea (China), transcriptomics demonstrated that in low oxygen concentrations, defined between 2 and 7 mg L^−1^ of oxygen, the transcripts of RuBisCO shifted from photoautotrophs to chemoautotrophs (Han et al., 2022). In addition, the coastal ecotypes of the ammonia-oxidizing *Thaumarchaeota* showed resilience to low-oxygen aquatic environments, with populations increasing along with the enhanced expression of core genes representing ammonia oxidation, ammonia transport, and carbon fixation (3-hydroxypropionic acid/4-hydroxybutyric acid cycle) pathways.

The bioavailability of dissolved organic matter (DOM) is important to biogeochemistry cycles with labile compounds being easily degraded and recalcitrant DOM more resistant to degradation by planktonic heterotrophic prokaryotes (Hansell et al., 2012; Hansell, 2013; Carlson and Hansell, 2014). The availability and decomposition of DOM have been studied in several OMZs (Devol and Hartnett, 2001; Keil et al., 2016; Le Moigne et al., 2017; Igarza et al., 2019; Lønborg et al., 2020). However, the bioavailability of DOM preserved in these OMZs has only been studied in a few experiments (Pantoja et al., 2009; Maßmig et al., 2019). Environmental studies in the Arabian Sea and coastal Pacific, have shown decreased organic matter remineralization under anoxic compared to oxic conditions (Devol and Hartnett, 2001; Keil et al., 2016). In contrast, the low organic matter remineralization rate observed in the coastal Chilean OMZ (Pantoja et al., 2009), was hypothesized to be related to the quality of the organic substrates rather than oxygen concentration. Degradation experiments in the Baltic Sea showed that oxygen plays a role in regulating particulate organic carbon degradation (Le Moigne et al., 2017), while a study using multifactorial batch experiments of microbial communities from the central Baltic Sea showed that the bacterial turnover of organic matter was limited by the availability of glucose and ammonium under both oxic and suboxic conditions (Maßmig et al., 2019).

In the present study, a microbial remineralization experiment was conducted to assess the bioavailability of DOC that accumulates under suboxic conditions and to determine how the microbial community responded to the reoxygenation of suboxic waters; thus, experimentally simulating a water column overturn event. Using prokaryotic (bacteria and archaea) abundance, DOC measurements, prokaryotic community changes, nutrient concentrations and DOM composition, these experiments by mimicking mixing emphasize the fate of carbon fixed under suboxic conditions and how the prokaryotic communities shift once water is reoxygenated. Furthermore, the results can be put into the context of the common mechanisms captured in the overturn system sampled *in situ* at Devil’s Hole.

## Methods

### Environmental sample collection

Samples were gathered biweekly aboard the R/V *Sea Dance* at the Devil’s Hole site in Harrington Sound, Bermuda (32°19.399’ N 64°43.08’ W) between May and October 2018. Continuous *in situ* profiling of dissolved oxygen (DO), salinity, pH, and temperature were conducted with a tethered YSI 556 multiprobe (Yellow Springs, OH, United States). Seawater samples from eight depths (1, 5, 10, 15, 20, 22, 23, and 24 m) were collected using a 5 L Niskin bottles attached to a hydro wire. Samples were collected and analyzed for concentrations of DO, dissolved inorganic carbon (DIC), total alkalinity (TA) and salinity to calibrate the YSI as described previously (Andersson et al., 2007; Bates, 2017). Nutrients, DOC, and prokaryotic abundance sampling was consistent with the best practices guide used for the GO-SHIP and BATS programs (Bates and Johnson, 2020; Halewood et al., 2022). Prokaryotic abundance samples were also used for fluorescent *in-situ* hybridization (FISH) and catalyzed reporter deposition (CARD)-FISH (Parsons et al., 2015). Bacterial production samples were collected in 40 mL polycarbonate vials, sealed, and placed into small coolers with water from the depth sampled to maintain the *in-situ* temperature until further processing following ^3^H-leucine incorporation method (Simon and Azam, 1989). Since suboxic conditions were not maintained, ^3^H Leu uptake rates maybe higher than actual *in-situ* rates. Genomic DNA samples consisted of 1 L of seawater being collected into polycarbonate bottles and concentrated onto Supor^®^ PES membrane filters (pore size 0.2 μm, dia. 47 mm, PALL Corporation, Albany, NY, United States). Each filter was preserved with 1 mL of sucrose lysis buffer (10 mM Tris.HCl; 10 mM EDTA; 300 mM NaCl; 0.75 M sucrose), sealed in a 3 mL cryovial, and stored at −80°C until further processing.

### Experimental design and seawater collection

Transplant experiments used to mimic mixing were conducted where surface and deep assemblages were inoculated into surface and deep 0.2 μm filtrate (media) to investigate the microbial response from a stratified water column to an overturn (reoxygenation) event. Seawater was collected from the Devil’s Hole site at oxic surface (1 m) and suboxic deep (23–24 m) water on August 9th, 2018, using a 10 L Niskin bottle. The 10 L niskin bottle used to collect water for the experiment was 1 m in length, with sampling depth determined by line payout. Thus, deep water sampling likely included water that spanned 22–24 m. Three experimental treatments were prepared (Table 1) by diluting whole (unfiltered) seawater by 70% with 0.2 μm seawater filtrate (Carlson and Ducklow, 1996) from different depths. The inoculum and filtrate were mixed under oxic conditions within a 12 L polycarbonate carboy and transferred to duplicate 5.5 L polycarbonate biotainer carboys (Nalgene, Rochester NY, United States). The three treatments included surface microbial assemblages mixed with filtrate from the suboxic deep waters or deep filtrate (S/D), deep microbial assemblages mixed with oxic surface filtrate (D/S) and surface assemblages mixed with oxic surface filtrate as a control (S/S). Surface assemblage treatments (S/S and S/D) were placed into a dry incubator (Fisherbrand Isotemp, Thermo Fisher, Waltham, MA, United States) and incubated in the dark at *in situ* temperature of the inoculum (30.0°C) for 21 days. The deep assemblage treatments (D/S) were incubated in the dark at an *in-situ* temperature of 21.8°C for 21 days. Samples for prokaryotic abundance and DOC concentration, FISH or CARD-FISH, DNA, total combined dissolved amino acid (TDAA), and DOM composition (high-resolution DOM, HR-DOM) were drawn throughout the incubation. All plasticware was soaked with 10% HCl and flushed with Milli-Q water before use.

**Table 1:** Experimental set-up including bottles, mixture volumes in % and liters, water source for microbial inoculum and 0.2 μm filtrate, sample depth, prokaryotic abundance, and DOC concentration at the time of sampling and measured at the start of the experiment.

Treatment	S/S	S/D	D/S
Bottles	A, B	C, D	E, F
Bottle volume (L)	5.5 L	5.5 L	5.5 L
Inoculum (volume/bottle)	Surface 30% (1.65 L)	Surface 30% (1.65 L)	Deep 30% (1.65 L)
0.2 μm filtrate (volume/bottle)	Surface 70% (3.85 L)	Deep 70% (3.85 L)	Surface 70% (3.85 L)
Sample depth	S = 1-2 m	S = 1–2 m; D = 22–23 m	S = 1–2 m; D = 22–23 m
Prokaryotic abundance (10^8^ cells L^−1^)	S = 17.6 ± 1.1	S = 17.6 ± 1.1	D = 70.0 ± 2.5
DOC conc μmol C L^−1^	100% S = 94.8 ± 2.4	70% D = 110.1 ± 4.7 30% S = 94.8 ± 2.4	70% S = 94.8 ± 2.4 30% D = 110.1 ± 4.7
Exponential growth duration (d)	10.1 ± 0.3	4.9 ± 0.0	2.8 ± 0.3
Specific Growth Rate μ d^−1^	0.03 ± 0.00	0.19 ± 0.11	0.11 ± 0.01
Change in prokaryotic cells ×10^8^ cells L^−1^ in exponential phase	8.1 ± 1.2	55.5 ± 3.5	24.9 ± 3.4*
DOM removal in exponential phase μmol C L^−1^	2.1 ± 0.3	7.1 ± 1.6	4.4 ± 1.6*
BGE % (using 20 fg C/cell)	7.9 ± 2.1	17.9 ± 9.4	12.1 ± 3.9

Calculated parameters include the time of exponential growth in days, the specific growth rate μ (d^−1^), the mean prokaryotic production over incubation in cells L^−1^ and the mean DOC removed over in μmol C L^−1^ used to determine the bacterial growth efficiency (BGE) in the exponential growth phase. *Days 0 and 0.63 were removed from the integration calculation as these were considered an initial death phase for the D/S treatment.

Experiment sampling was carried out using a custom positive pressure system that enabled subsampling without removing caps from the biotainers in order to reduce DOC contamination (Baetge et al., 2021). An aquarium pump pumped air through a hydrocarbon trap, which pressurized the biotainers and displaced sample water through submerged Teflon tubing into collection bottles to reduce sample handling (Liu et al., 2020). The YSI 556 multiprobe (Yellow Springs, OH, United States) was used to keep track of temperature (°C), salinity (ppt), pH, and dissolved oxygen (mg L^−1^ converted to μmol L^−1^) over 24 h and again on day six6 by collection of ~50 mL in a 100 mL polycarbonate beaker. Prokaryotic abundance and FISH or CARD-FISH samples (40 mL) were collected as described by Parsons et al. (2015). Nutrient samples were collected into 60 mL acid-washed high-density polyethylene (HDPE) bottles and stored at −20°C. DOC samples (duplicate 30 mL aliquots) were filtered through 0.2 μm polycarbonate filters (Millipore, Burlington, MA, USA; prewashed in 10% HCl for 1 h and rinsed in sterile water), packed in 25 mm Swinnex filter holders attached directly to sample line with Luer lock adaptor, and collected into 40 mL pre-combusted EPA glass vials with polytetrafluoroethylene (PTFE) coated silicone septa. DOC samples were acidified with 50 μL 4 N HCl to a pH of 3. TDAA samples were filtered through the same filters into 60 mL acid-washed high-density polyethylene (HDPE) bottles and stored at −20°C. The low-volume filters were retained for DNA analysis and were stored at −80°C until DNA extraction. In addition, 1 L of seawater was filtered through an Omnipore PTFE filter (pore size 0.2 μm, dia. 47 mm, Millipore, Burlington MA, United States) housed in a perfluoroalkoxy (PFA) filter holder for the analyses of organic metabolites via ultra-high resolution mass spectrometry at three timepoints during the 21- day experiment corresponding to day 0, day 8, and day 21. The filtrate (1 L) collected into a PFA bottle for organic metabolites was acidified with 1 mL ultrapure HCl (Optima, 35%, Thermofisher Scientific, Waltham, MA, United States) to pH ~3, stored at 4°C and extracted within 24 h for mass spectrometry analysis. The filters were retained for high-volume DNA analysis and were stored at −80°C until DNA extraction.

### Chemical analyses (DO, salinity, DIC, TA, and nutrients)

Salinity was analyzed using an auto salinometer according to the Bermuda Atlantic Time-series Study (BATS) methodology (Knap et al., 1997; Bates and Johnson, 2020). Seawater DO was determined by automatic Winkler titrations based on a UV end-point detector system according to the BATS protocol (Knap et al., 1997; Bates and Johnson, 2020). DIC and TA were analyzed according to Andersson et al. (2007). DIC parameters pCO_2_ and pH_TOT_ (pH defined on a total H+ scale) were calculated at *in situ* temperature and salinity conditions based on TA and DIC data (Andersson et al., 2007). Nutrient chemistry, including total ammonium (ammonia plus ammonium), nitrite, nitrite plus nitrate, ortho-phosphate, and silicic acid analytes, was performed via flow-injection analysis on a Lachat QuickChem 8,500 Series 2 by the University of California, Santa Barbara Marine Science Institute Analytical Laboratory.^1^

### DOM analyses (DOC, TDAA, HR-DOM)

Replicate DOC samples were analyzed using a high-temperature combustion method (Halewood et al., 2022) on a modified TOC-V or TOC-L analyzer (Shimadzu, Kyoto, Japan) at the University of California, Santa Barbara. All DOC samples were analyzed with a set of “in-house” reference waters that were previously calibrated with DOC Certified Reference Material (CRM) provided by D. Hansell (University of Miami) (Halewood et al., 2022). The precision for DOC analysis is ~1 μmol L^−1^ or a CV of ~2%. DOC values greater than the 90th percentile were considered contaminated.

Replicate TDAA samples were hydrolyzed by 6 N HCl (with 1% 12 mmol L^−1^ ascorbic acid to prevent oxidation of amino acids by nitrate) under nitrogen at 110°C for 20 h (Parsons et al., 1984; Henrichs, 1991; Kuznetsova and Lee, 2002). Hydrolysate was filtered through combusted quartz wool and neutralized via evaporation under nitrogen. Nanopore blanks followed the same extraction protocol as samples. Amino acids were analyzed by high-performance liquid chromatography (HPLC, Dionex ICS 5000+) equipped with a fluorescence detector (Dionex RF2000, Ex = 330 nm, Em = 418 nm) after pre-column *o*-phthaldialdehyde derivatization (Lindroth and Mopper, 1979; Lee et al., 2000; Kaiser and Benner, 2009; Liu et al., 2013).

Acidified 0.2 μm filtered seawater samples were passed through Bond Elut PPL cartridges (1 g/6 mL; Agilent, Santa Clara, CA, United States) to extract extracellular DOM. Extraction followed the protocol (Dittmar et al., 2008) with modifications described in Longnecker (2015). The extraction efficiency for marine DOC using PPL cartridges is 43–62%. Extracted DOM samples were analyzed in negative ion mode using untargeted mass spectrometry methods (Weber et al., 2022) at the Woods Hole Oceanographic Institution. Mass-to-charge (*m/z*) ratios, retention times, and peak areas were measured for each sample. mzRT features were defined as unique combinations of an *m/z* ratio and a retention time. Elemental formulas were calculated from *m/z* ratios using the Compound Identification Algorithm (Kujawinski and Behn, 2006).

### Prokaryotic abundance

Fixed seawater (1–5 mL) was filtered onto 0.2 μm polycarbonate filters pre-stained with Irgalan Black (0.2 g in 100mLs of 2% acetic acid). Prokaryotic abundance (bacteria and archaea) was determined by staining with 0.5 mL of 4′,6-diamidino-2-phenylindole dihydrochloride (DAPI; Sigma-Aldrich, St. Louis, MO, United States) (5 μg mL^−1^) for 3 min (Porter and Feig, 1980). Ultraviolet epifluorescence microscopy (AX70; Olympus, Shinjuku, Japan) excited the stained prokaryotic cells for counting. At least 10 fields of view containing 40–100 prokaryotic cells were enumerated for each sample at a magnification of ×1,000. Narrow green epifluorescence microscopy (AX70; Olympus, Shinjuku, Japan) was used to excite the autotrophic cells (*Synechococcus* in this study) for counting. At least 10 fields of view containing 0–25 autotrophic cells were enumerated for each sample at a magnification of ×1,000.

### Archaeal and bacterial lineage abundance using FISH and CARD-FISH

FISH was used to enumerate *Chlorobi* and SAR202 using probes Chlorob441-Cy3 and SAR202_103R-Cy3 & 311R-Cy3 with Neg338 as a negative control (Integrated DNA Technologies, Coralville, IA, United States). Ten to forty milliliter of fixed seawater samples were filtered onto 0.2 μm polycarbonate membrane filters (Osmotics, Norfolk, United Kingdom) under gentle vacuum (100 mmHg) and stored at −20°C with desiccant. Filters were divided into eight sections, and each section was washed in 95% ethanol and then probed according to (Parsons et al., 2015; Liu et al., 2020).

CARD-FISH (Teira et al., 2004; Herndl et al., 2005) was used to enumerate *Thaumarchaeota* using probe Cren537 and NON338 (non-specific) as a negative control (Integrated DNA Technologies, Coralville, IA, United States). For *Thaumarchaeota*, no embedding step was performed prior to incubation in hydrochloric acid (0.1 N) for 2 min to permeabilize the cell membrane (Parsons et al., 2015). The hybridization and wash conditions with all probe sequences described in (Parsons et al., 2015). A tyramide signal amplification (TSA) kit was used after hybridization to improve fluorescence and probe detection (PerkinElmer, Waltham, MA, United States).

The resulting filters from FISH and CARD-FISH were mounted with 20 μL of 1.67 μg ml^−1^ 4′,6-diamidino-2-phenylindole dihydrochloride (DAPI; SIGMA-Aldrich, St. Louis, MO, United States) in Citiflour solution (Ted Pella Inc., Redding, CA, United States), sealed with nail polish, and stored in the dark at −20°C. Imaging was performed with an Olympus AX70 epifluorescent microscope on FISH and CARD-FISH slides excited with Cy3 (550 nm) and UV wavelengths, as previously described (Parsons et al., 2012, 2015). The image capturing was performed using a Color Retiga Exi (QImaging^®^, Surrey, BC, Canada) digital camera (1,392 × 1,040 pixels) with Image Pro software (version 7.0; Media Cybernetics, Rockville, MD, USA) (Carlson et al., 2009).

### DNA extraction, library preparation, and Illumina sequencing

DNA was extracted using the phenol-chloroform protocol (Giovannoni et al., 1990). After drying, the DNA pellet from the high-volume samples was shipped to the Center for Genome Research and Biocomputing (Oregon State University), Corvallis, Oregon for sequencing. Genomic DNA samples were amplified using the universal primer sets for the 16 s ribosome V4 region 515F (GTGCCAGCMGCCGCGGTAA) and 806RB (GGACTACNVGGGTWTCTAAT) with ‘general’ Illumina overhang adapters (Apprill et al., 2015). Libraries were pooled in equimolar concentrations prior to sequencing. Samples were sequenced using one 2 × 250 Paired-End lane with a MiSeq Reagent Kit v2.

The DNA pellet from low-volume samples was shipped to the University of California, Santa Barbara. Amplification of the V4 region of the 16S rRNA gene was performed using the 515F-Y (5′-GTGYCAGCMGCCGCGGTAA-3′) and 806RB (5′-GGACTACNVGGGTWTCTAAT-3′) primers with custom adapters (Apprill et al., 2015; Parada et al., 2016; Wear et al., 2018). PCR-grade water process blanks and mock communities (BEI Resources mock communities HM-782D and HM-783D) and a custom community from the Santa Barbara Channel (Wear et al., 2018) were included with each 96-well plate of samples as quality control checks. Amplicons were cleaned and normalized using SequalPrep plates (Invitrogen, Waltham, MA, United States), pooled at equal volumes, concentrated using Amicon Ultra 0.5 mL centrifugal tubes (Millipore, Burlington, MA, United States), gel extracted using the QIAquick Gel Extraction Kit to remove non-target DNA (Qiagen, Hilden, Germany) and sequenced on an Illumina MiSeq using PE250 chemistry at University of California (UC), Davis DNA Technologies Core.

### Archaeal *amoA* and prokaryotic *cbbL* gene quantification using qPCR

Quantitative PCR (qPCR) was performed on an ABI 7300 Real Time PCR machine (Thermo Fisher Scientific, Waltham, MA, USA) using 7300 System SDS Software v1.2 (Applied Biosystems). The *cbbL* gene abundance was investigated since it encodes for the ribulose-1,5-bisphosphate carboxylase/oxygenase (RuBisCO) form I enzyme responsible for the first step in carbon fixation. The *cbbL* gene is present within lineages found within Devil’s Hole, including *Synechococcus* and *Chlorobiales* (Pichard et al., 1996; Eisen et al., 2002; Parsons et al., 2015). Primers were designed in this study for the *cbbL* gene as described in Supplementary Table S1. The *amoA* gene encodes for the ammonia monooxygenase enzyme that converts total ammonium to nitrite. Since *Thaumarchaeota*, a known ammonia oxidizing archaea (AOA) is found in Devil’s Hole (Parsons et al., 2015) and has a higher affinity for ammonia than competing bacteria, the *amoA* gene abundance was also quantified in this study using the primer set from (Treusch et al., 2005).

The *cbbL* and *amoA* gene abundance was performed in duplicate on the undiluted and diluted (20 ng/μL) samples. Each reaction mixture consisted of 10 μL of Luna Universal qPCR Master Mix (New England Biolabs, Ipswich, MA, United States), 10 mM of each forward and reverse primer, 2 μL of template and 6 μL of sterile water (New England Biolabs, Ipswich, MA, United States). All reactions were performed in optical qPCR tubes with strip caps (USA Scientific, Ocala, FL, United States). Negative controls (containing nuclease-free water in replace of the template DNA) were analyzed with every experiment. Samples of interest were amplified using the thermal conditions outlined in Supplementary Table S2 (PCR efficiency *cbbL* = 119%; *R*^2^ = 0.994; PCR efficiency *amoA* = 119%; *R*^2^ = 0.998). Standards were prepared for the *cbbL* and *amoA* genes as described in Supplementary Table S2. The gene copy number (gcn) of the standard was calculated using the following formula (Prediger, 2013):


GCN=DNAconc(ng)×6.0221×1023(molecules/mole)StandardLength(bp)×1×109(ng/g)×660(g/mole)

The gcns of the genes were then divided by the ng concentrations of DNA in the sample to normalize the qPCR data. The standards were diluted from 10^6^ to 10^1^ gene copies in all experiments, and a standard curve was subsequently created.

### Data processing and analysis

Ocean Data View version 5.5.2 (Schlitzer, 2021) was used to create contour plots using the DIVA (Data-Interpolating Variational Analysis) gridding algorithm (Barth et al., 2010). Statistical analyses were conducted using base R version 4.1.2 and R Studio 2021–09.1 (Team, 2021). Prokaryotic specific growth rates (μ) were calculated from experimental growth curves as the slope of ln(PA) vs. time during the exponential growth phase of each treatment (Table 1). Stationary phase was reached at different times for each treatment and was determined as 2 × t_mid_ where t_mid_ is the time when PA reached half carrying capacity using a logistic growth model in the growthcurver package in R (Sprouffske, 2018). DOC concentrations at the calculated stationary time point were interpolated if sampling did not coincide with the modeled estimate of stationary phase. Mean prokaryotic abundance growth and DOC drawdown over exponential growth phase were derived from integration under the growth curve or DOC curve and normalized over incubation time (Table 1). Bacterial growth efficiency (BGE) was calculated as the ratio between bacterial carbon (BC) increase and DOC removal from T0 to stationary phase (Liu et al., 2020):


$$BGE=\frac{\int_{T0}^{Tstationary}BC\;dt}{\int_{T0}^{Tstationary}DOC\;dt}$$

where t is time, ∫BC is the time normalized integrated prokaryotic growth converted to carbon unit assuming a conversion factor of 20 fg C per cell (Lee and Fuhrman, 1987) and ∫DOC is the time normalized integrated DOC draw down over exponential phase.

TDAA yield was calculated as the sum of TDAA in carbon units normalized by DOC concentration. Degradation index (DI) was calculated based on Dauwe et al. (1999) and Kaiser and Benner (2009):


$$DI=\sum \frac{{\mathrm{var}}_{i}-AVG{\mathrm{var}}_{i}}{STD{\mathrm{var}}_{i}}*fac.coef_{i}$$

Where var_i_ is the molar percentage of amino acid i, and AVGvar_i_, STDvar_i_, and fac.coef_i_ are average value, standard deviation, and principal component factor coefficient of amino acid i derived from a variety of DOM samples described in Kaiser and Benner (2009).

The mzRT features were grouped into compound classes based on the elemental formulas and the calculated aromaticity index (Koch and Dittmar, 2016). The following groups were defined: black carbon, carbohydrates, condensed hydrocarbons, highly unsaturated compounds, lignin, lipids, peptides, proteins, protein maya, polyphenols, unsaturated aliphatic compounds, sugars, saturated fatty acids, and carboxyl-rich alicyclic molecules (CRAM) (Martínez-Pérez et al., 2017). From the molecular formula assignments, the magnitude-averaged O/C, H/C, and double bond equivalent (DBE) values for each sample were calculated for each assigned molecular formula based on the relative magnitude of each peak (Sleighter and Hatcher, 2008).


$$DBE=\frac{1}{2}\left(2+2C-H+N+P\right)$$

Sequence data were trimmed, dereplicated, checked for chimeras, and assigned to taxonomies using the DADA2 R package, v1.2 (Callahan et al., 2016) and the SILVA SSU Ref database version 138 (Glockner et al., 2017). Illumina sequencing data was filtered to remove amplicon sequence variants (ASVs) in less than four samples and rarefied to a minimum of 10,000 reads. Stacked bar charts were plotted for the most abundant (>0.15%) ASVs using phyloseq and ggplot 2 packages in R.

## Results

### Stratification, the formation of the Devil’s Hole OMZ, and subsequent overturn in 2018

**Figure 1:**
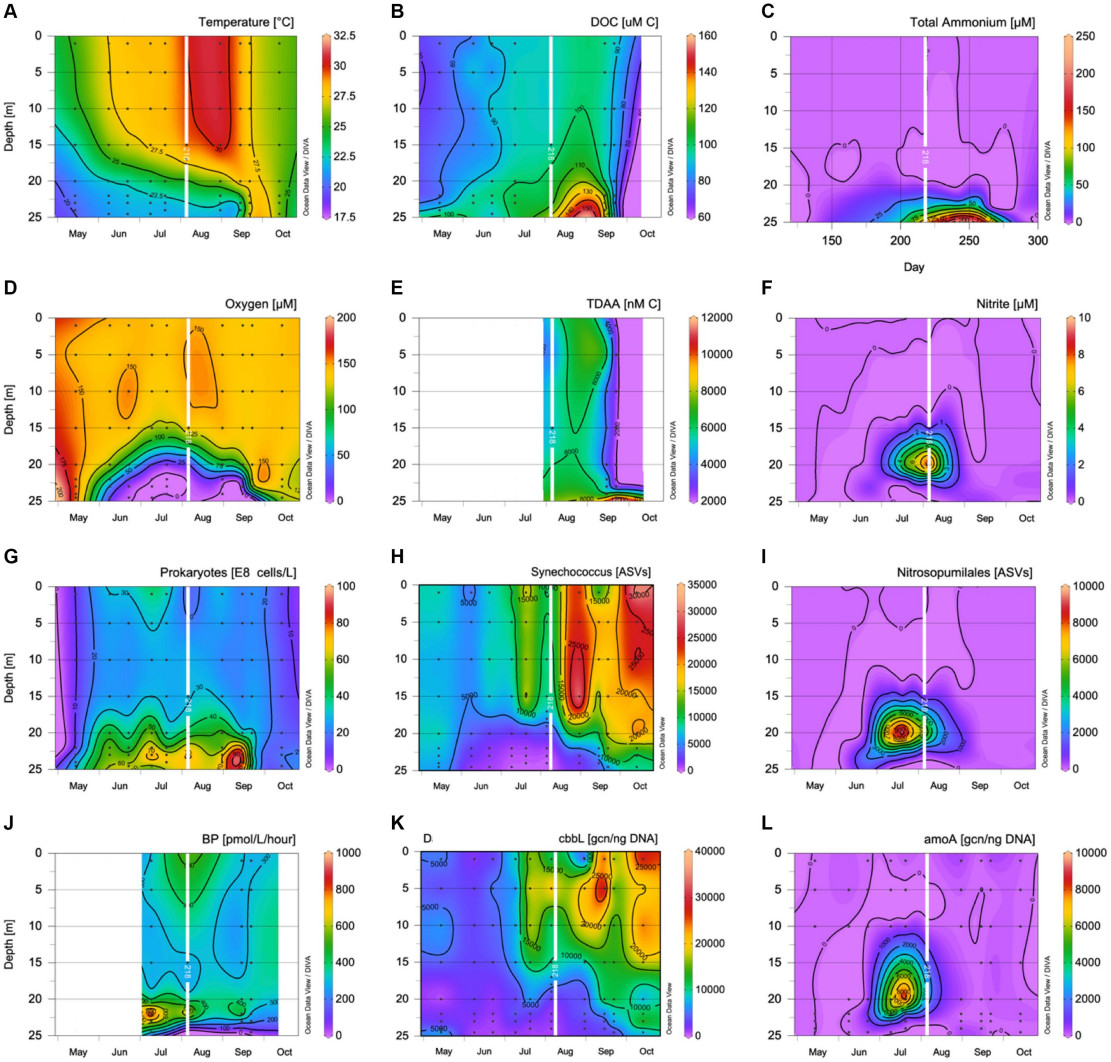
Contour plots showing **(A)** temperature (°C); **(B)** dissolved organic carbon concentrations (μmol C L^−1^); **(C)** total ammonium concentrations (μmol L^−1^); **(D)** dissolved oxygen concentrations (μmol L^−1^); **(E)** TDAA concentrations (nmol L^−1^); **(F)** nitrite concentrations (μmol L^−1^); **(G)** prokaryotic abundance (10^8^ cells L^−1^); **(H)** *Synechococcus* ASV number; **(I)** *Nitrospumillales* ASV number; **(J)** ^3^H Leu uptake (pmol/L/h); **(K)** cbbL gene copy number per ng of DNA; and **(L)** amoA gene copy number per ng of DNA. The day of seawater collection for the experiment in August is overlayed as a white line.

The water column began to stratify in May 2018 with a 5°C gradient from surface to bottom. Bottom waters (22–24 m) ranged between 20°C and 22°C between May 16th and July 23rdJuly23rd, 2018. By June 11th, 2018, oxygen concentrations in the bottom waters were reduced to 26 μmol L^−1^, and were within the dysoxic range and reduced to suboxic levels (<20 μmol L^−1^) by July 12th, 2018 (Wright et al., 2012). The temperatures in the bottom waters started to warm on July 23rd, ranging between 22°C and 24°C until September 19th, 2018. Partial overturn occurred between September 19th and 26th, 2018 (Figure 1). During this time, temperature was uniform at 27.6°C throughout the water column, while oxygen concentrations remained suboxic at 24.5 m (Figure 1), which was just above the sediment layer. Complete overturn occurred by October 18th, 2018, with all parameter profiles returning to uniform distributions.

### The spatial changes in biogeochemical and microbial variables within devils hole, Bermuda, at the time of experimental sampling

On August 9th, 2018, the temperature difference between surface (30°C) and deep depths (21.8–22.4°C) was 8.2°C. Oxygen concentrations decreased from surface 148 μmol L^−1^ to suboxic levels of 1–2 μmol L^−1^ from 22 m through 25 m. The surface prokaryotic abundance was 17.6 ± 1.1 × 10^8^ cells L^−1^, while the surface DOC concentration was 94.8 ± 2.4 μmol C L^−1^. From 22 m through 25 m, prokaryotic abundance was elevated ~70 × 10^8^ cells L^−1^, and DOC concentrations ranged between 105 and 115 μmol C L^−1^ (Figure 1; Table 1). Bacterial 3H -Leu incorporation rates reached a maximum in the oxycline with rates of >800 pmol L^−1^ h^−1^ at 22 m, almost double the 3H -Leu incorporation rate measured at the surface, decreasing to 75 pmol L^−1^ h^−1^ at 24 m. TDAA C concentrations reached 6,742 nmol C L^−1^ at 24 m while degradation indices ranged from 2.1 to 2.4 throughout the water column (Figure 1; Supplementary Table S4). Nitrate levels were below the detection limit for the whole water column, while the other nutrients all increased with depths with concentrations for phosphate, silicate, nitrite, and total ammonium reaching 1.8, 66.3, 0.5, and 79.2 μmol L^−1^, respectively at 24 m. The gene copy number (gcn) normalized to DNA concentration per ng were 9922.7 ± 314.2 copies for the *cbbL* gene at the surface and 140.0 ± 58.3 copies between 22 and 24 m (Figure 1). The gene copy number (gcn) normalized to DNA concentration per ng were 14.1 ± 0.4 copies for the *amoA* gene at the surface, peaked at 20 m with 2.70 ± 0.09 × 10^4^ copies for the *amoA* gene, and averaged 1363.4 ± 374.9 copies between 22 and 24 m (Figure 1). There were few *Nitrospumillales* ASVs in the sequencing data at the surface, but *Synechococcus* ASVs were abundant and coincided with the *cbbL* gene copy number (Figure 1). *Nitrospumillales* ASVs peaked at 20 m concurrent with the *amoA* gene copy number peak, while *Synechococcus* ASVs were low, decreasing with depth, and followed a similar pattern to the *cbbL* gene copy number (Figure 1).

### Prokaryotic abundance and DOM dynamics in the experimental treatments

In the initial inoculum mixed with filtered water, the prokaryotic assemblages were 50% higher in the S/S and S/D treatments than expected, suggesting smaller cells may have filtered through while the D/S treatment was within the 20% error. Actual DOC concentrations were within 2–5% of the expected DOC concentrations based on conservative mixing calculations (Table 1). The suboxic deep DOM was not kept under suboxic conditions during the experimental set-up and will be referred to as deep DOM during the experimental descriptions. In fact, oxygen levels increased to 98 μmol L^−1^ in the S/D treatment and 116 μmol L^−1^ in the D/S treatment within 15 h as determined using YSI 556 multiprobe (Yellow Springs, OH, United States).

**Figure 2:**
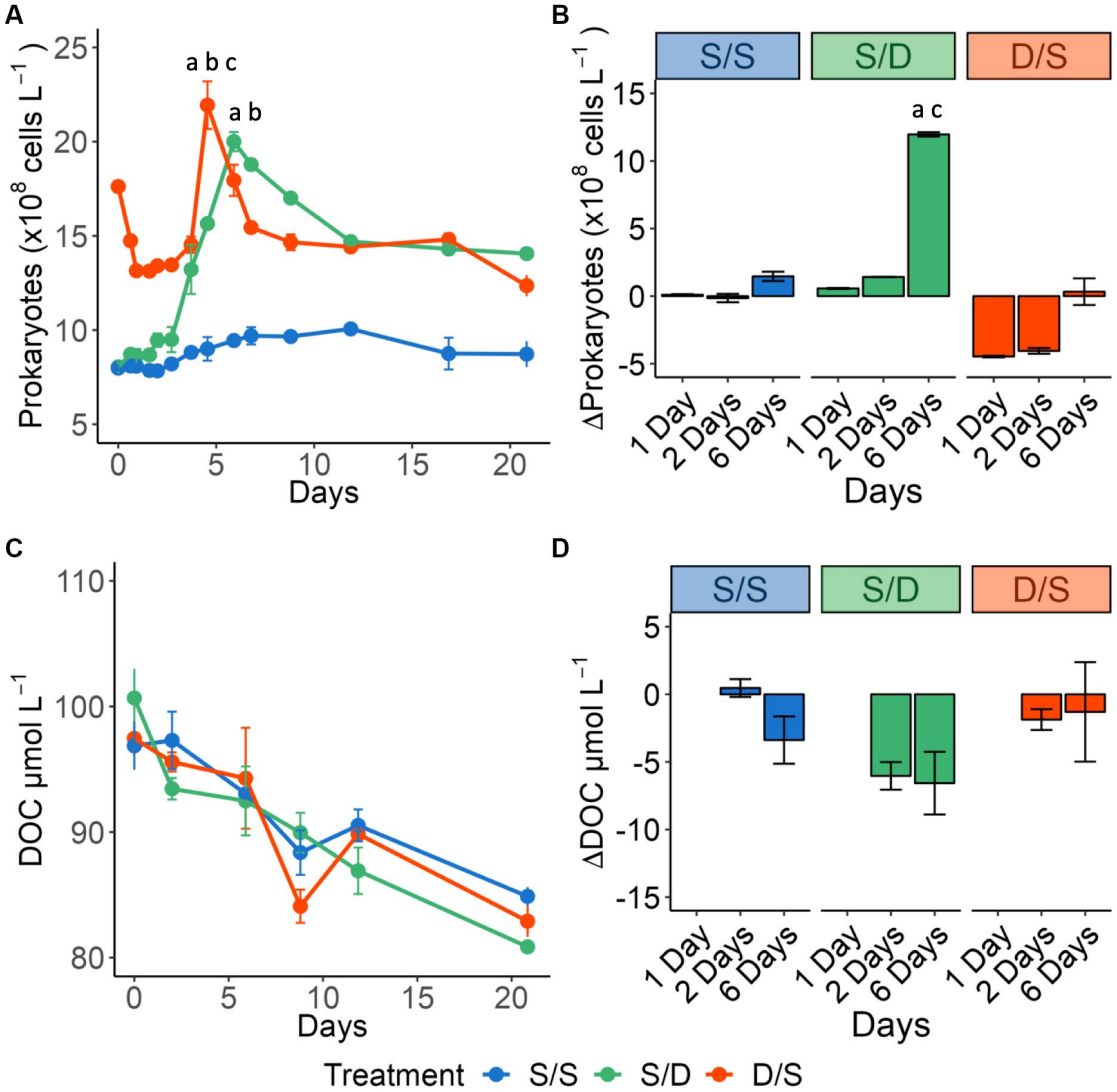
Line graphs showing **(A)** prokaryotic cell abundance (×10^8^ cells L^−1^) for all treatments over 21 days. Significant differences between treatments on day 7 and day 8 are indicated by “a” for the S/S vs. S/D treatments, “b” for the S/S vs. D/S treatments and “c” for the S/D vs. D/S treatments. Bar plots showing the change from day 0 for **(B)** prokaryotic cell abundance (×10^8^ cells L^−1^) for Days 1, 2, and 6 measured within the exponential growth phase. Significant differences between treatments on day 6 are indicated by “a” for the S/S vs. S/D treatments and “c” for the S/D vs. D/S treatments. Line graphs showing **(C)** DOC concentrations (μmol C L^−1^). Bar plots showing the change from day 0 for **(D)** DOC concentrations (μmol C L^−1^) for Day 2, and Day 6 measured within the exponential growth phase.

The prokaryotic response was distinct for each treatment (Figure 2A), with no significant differences between replicate bottles (*p* = 0.551, *N* = 42). The S/S treatment had the lowest specific growth rate and prokaryotic growth during exponential phase (Table 1). The S/D treatment had the highest specific growth rate, more than a sixfold increase from the S/S control, and the highest prokaryotic growth during exponential phase (Figure 2B). The prokaryotic abundance initially decreased in the D/S treatment, followed by rapid exponential growth phase between day 2 and day 5, with a specific growth rate between the other two treatments. Prokaryotic abundance in the two treatments was significantly different from the control (ANOVA and Tukey HSD *p* < 0.01). The S/D treatment had the highest BGE of all the treatments, with values more than twice that of the control, while the D/S treatment had BGE values 1.5 times that of the control.

DOC removal had no significant difference between replicate bottles (*p* = 0.232, *N* = 18). During the 2-day lag phase, DOC concentrations did not change in the S/S treatment but then decreased at a rate of 0.79 μmol C L^−1^ d^−1^ over the remaining 19 days (Figure 2C). In the S/D treatment, DOC was drawn down quickly, with concentrations decreasing by 2.7 μmol C L^−1^ d^−1^ over the first 2 days (Figure 2D) and then by 0.64 μmol C L^−1^ d^−1^ over the remaining 19 days. In the D/S treatment, DOC concentrations decreased by 0.68 μmol C L^−1^ d^−1^ over the 21-day experiment, with a small increase of DOC concentration between day 9 and day 12. Over the 6 days when prokaryotic abundance peaked, S/D had the largest DOC removal among the three treatments; similarly, mean DOC removal over exponential phase in S/D was over 3-fold greater than in the S/S treatment while DOC removal in the D/S treatment was double that in the S/S treatment (Figure 2D; Table 1). From Days 6 to 21, DOC concentration in three treatments became close to each other (Figure 2C) with no significant differences between all treatments throughout the experiment (ANOVA and Tukey HSD *p >* 0.90).

### DOM quality change revealed from TDAA

**Figure 3:**
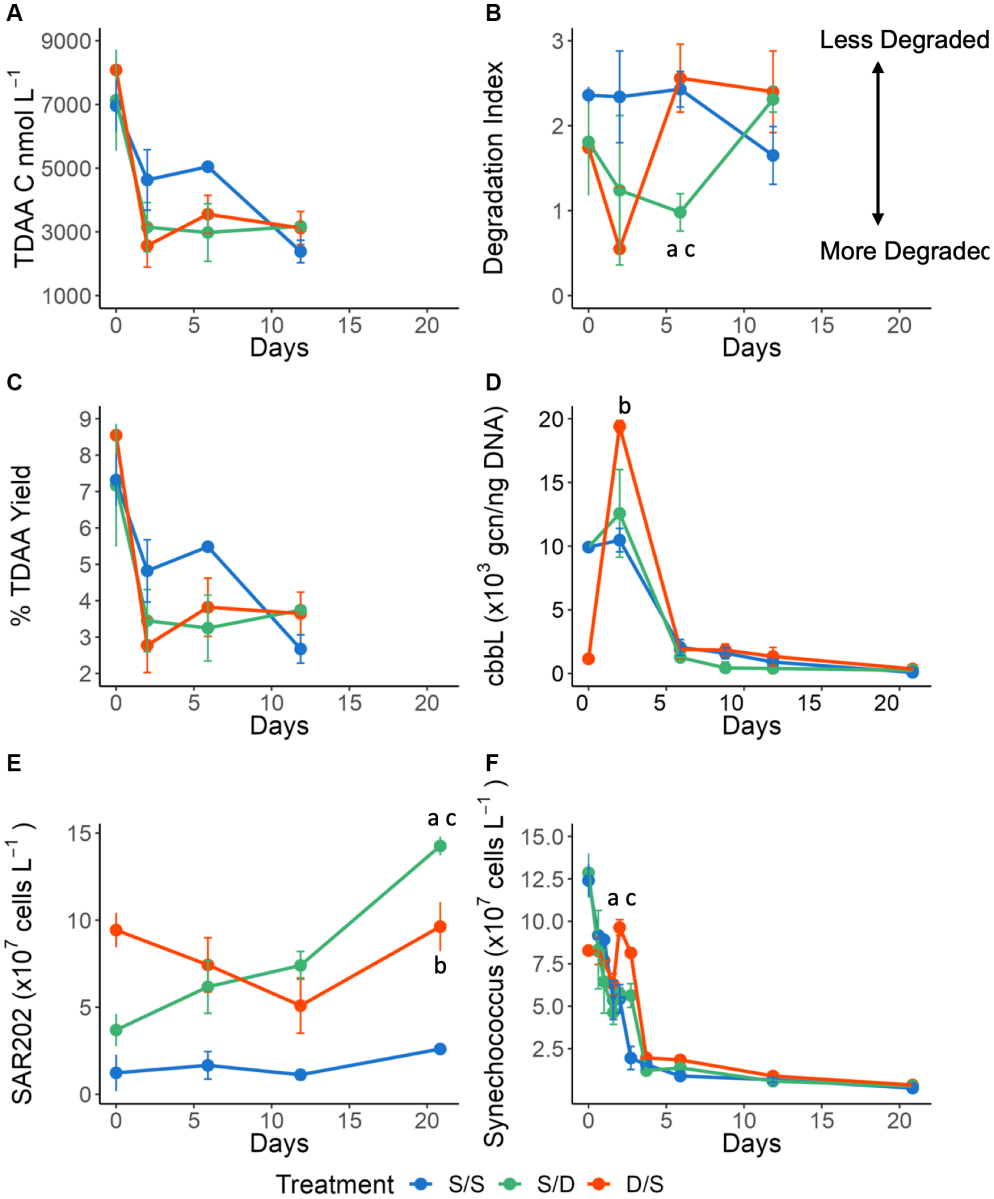
Line graphs showing **(A)** total dissolved amino acids (TDAA) concentrations in nmol C L^−^1; **(B)** the Degradation Index; **(C)** percent TDAA yield; **(D)** cbbL gene copy number per ng of DNA; **(E)** SAR202 cell abundance ×10^7^ cells L^−1^; and **(F)** *Synechococcus* cell abundance ×10^7^ cells L^−1^ for all treatments over 21 days. Significant differences between treatments on specific timepoints are indicated by “a” for the S/S vs. S/D treatments, “b” for the S/S vs. D/S treatments and “c” for the S/D vs. D/S treatments.

In addition to changes in bulk DOC concentrations, DOM quality also changed throughout the incubations, as revealed by the TDAA data. The initial TDAA concentrations were greatest in the deep waters compared to the surface during August and reached a maximum in the suboxic layer in September (Supplementary Table S3). For all treatments, the average TDAA-C concentration decreased over time, with the greatest removal rate observed in the D/S treatment (Figure 3A). As a diagenetic index of DOM, TDAA yield in each treatment decreased rapidly over the first 2 days of the incubation, indicating that as DOC was consumed, it became more diagenetically altered (Figure 3B). While TDAA yield in the S/S treatment continued to decrease through Day 12, it curiously increased from Days 6 to 12 in the S/D treatment and after Day 2 in the D/S treatment. Another diagenetic index, DI, is derived from amino acid composition. DI showed lower values of <1 with degraded, more refractory DOM considered to have a degradation index of <1 (Dauwe et al., 1999; Kaiser and Benner, 2005). Consistent with TDAA yield, DI in the S/D treatment decreased at a faster rate than the control, reaching a low after 6 days before increasing again by day 12 (Figure 3D). The decrease in DI in the D/S treatment, reaching the lowest value after only 2 days. After 2 days, the DI increased to its highest level in the D/S treatment, indicating a potential input of fresh DOM.

**Figure 4:**
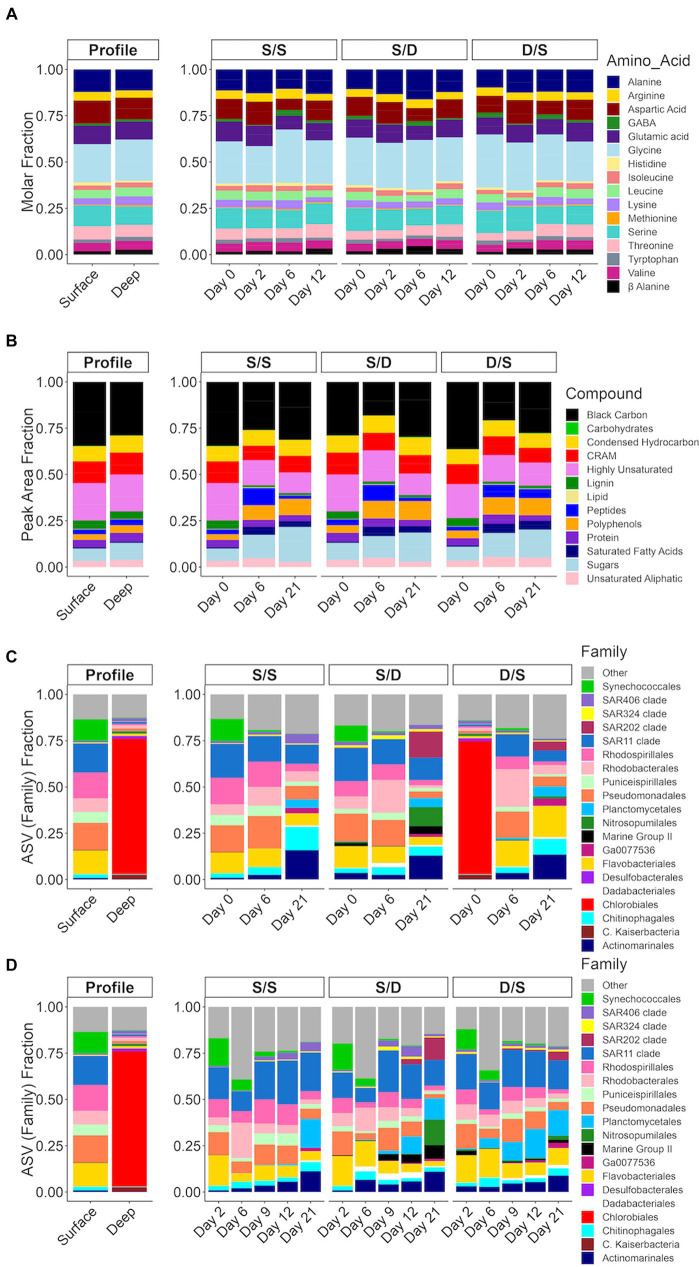
Bar plots for the treatments with **(A)** molar fraction of each amino acid; **(B)** the peak area fraction of the mzRT features in each of the defined compound classes (Martínez-Pérez et al., 2017); and the fraction of total Illumina sequencing amplicon sequence variants (ASVs) of the V4 region of the 16S ribosomal RNA gene for each of the dominant family groups divided by the total ASVs for **(C)** high-volume samples (1 L) filtered onto Omnipore filters and **(D)** low-volume samples (~200 mL) filtered onto polycarbonate filters. The ASVs belonging to family groups less than 1% of total are combined into “other”. In the mzRT features plot **(B)** S/S Day 0 and D/S Day 0 represents the surface DOM samples and S/D Day 0 represents the deep DOM samples.

The percent contribution of each amino acid to total amino acid concentrations were plotted as a stacked bar plot for the time series in August and September (Supplementary Figure S2) and for the experiment (Figure 4A). The percent molar concentrations of amino acids did not change much over depth in August. By September, glycine had reduced from 29% of the total amino acid concentrations at the surface to 17% in the suboxic layer, while glutamic acid increased from 8% at the surface to 14% in the suboxic layer (Supplementary Figure S2). The concentration of alanine increased from 86.95 nmol L^−1^ in the surface to 286.22 nmol L^−1^ at depth, while the percent contribution to total amino acids decreased from 13% at the surface to 11% at 24 m.

In the experiment, alanine, asparagine, glycine, glutamine, and serine have the highest concentrations representing between 62 and 76% of the total amino acid concentrations, depending on treatment and timepoint. Preferential removal of specific amino acids, such as glutamic acid, aspartic acid, serine, and leucine, occurred in the S/S, S/D and D/S treatments with 63, 45, and 38% of the amino acid composition in the fraction of TDAA removed, respectively over 6 days (Figure 4A). For instance, serine was removed at a rate double that of the control (155 nM compared to 68 nM) (Supplementary Figure S3). While the percent contribution of glycine to total amino acids increased by 8% between days 2 and 6 in the S/S treatment, it remained relatively constant between days 2 and 12 in the S/D treatment, followed by a 5% decrease between days 2 and 12 in the D/S treatment. The molar percentage of alanine was relatively constant in the control and D/S treatments but decreased by 3% from days 6 to 12 in the S/D treatment.

### mzRT feature composition in the experimental treatments

The metabolomic data showed similar mzRT feature numbers when comparing the initial surface to the initial deep DOM (Table 2). The magnitude-averaged ratios of H/C_w,_ a saturation index, increased over 8 days, showing a saturation decrease. This index then decreased slightly, showing increased saturation by day 21. The magnitude-averaged ratios of O/C_w_ increased over the experiment, indicating an increase in oxidative state. The magnitude-averaged values of DBE_w_ decreased over the experiment. The mzRT features defined by DOM compound class were grouped together and plotted as the corresponding percent peak area for each of the compound classes based on the elemental formulas and the calculated aromaticity index (Koch and Dittmar, 2016). Lignin was seen in both the surface DOM and deep DOM, while the number of black carbon mzRT features was elevated in the surface DOM compared to deep DOM (Figure 4B). In the experiment, peptides, polyphenols, and sugar classes contributed more to the total defined classes in all three treatments by day 6. In contrast, the black carbon class contribution to the total defined classes decreased in all three treatments by day 6. By the end of the experiment, the sugar class had increased by 10% of the total defined classes in all three treatments when compared to the start of the experiment. The fraction of carboxyl-rich alicyclic molecules (CRAM), a major fraction of refractory DOM (Hertkorn et al., 2006), decreased slightly by day 6 in the S/S and S/D treatments and by day 21 in the D/S treatment.

**Table 2:** Experimental data including metabolite information including the total number of mzRT features, the number of mzRT features with elemental formulas, and the number of mzRT features defined by DOM compound classes.

	Metabolites^1^								ASV diversity indices^2^		ASV sequencing reads^2^	
Sample ID	#mzRT features	#Elemental formulas	#CHO	#CHON	#CHOS	H/C_w_	O/C_w_	DBE_w_	Shannon Weiner	Simpson	Rep 1	Rep 2
Surface	5,456	4,573	1,737	1,724	353	0.64	0.37	6.01	4.39 ± 0.04	0.97 ± 0.00	79,132	
Deep	5,675	4,781	1,803	1,793	387	0.67	0.40	6.48	2.39 ± 0.08	0.85 ± 0.05	70,773	
S/S Day 0	5,456	4,573	1,737	1,724	353	0.64	0.37	6.01	4.33 ± 0.02	0.97 ± 0.00	85,786	101,742
S/S Day 6	6,162	5,192	1,919	1,928	420	0.94	0.45	5.94	4.51 ± 0.02	0.97 ± 0.00	77,800	120,255
S/S Day 21	6,006	5,047	1,843	1,876	414	0.75	0.52	5.98	4.21 ± 0.13	0.97 ± 0.01	90,621	77,456
S/D Day 0	5,675	4,781	1,803	1,793	387	0.67	0.40	6.48	4.89 ± 0.00	0.98 ± 0.00	104,462	98,281
S/D Day 6	6,216	5,229	1,930	1,944	433	0.97	0.43	6.31	4.61 ± 0.01	0.98 ± 0.00	99,000	145,880
S/D Day 21	6,077	5,835	2,150	2,181	468	0.76	0.49	6.01	3.97 ± 0.11	0.95 ± 0.01	71,393	116,116
D/S Day 0	5,456	4,573	1,737	1,724	353	0.64	0.37	6.01	2.39 ± 0.08	0.85 ± 0.05	89,512	70,773
D/S Day 6	6,048	5,087	1,865	1,904	416	0.92	0.45	6.12	4.80 ± 0.01	0.98 ± 0.00	62,568	84,747
D/S Day 21	6,031	5,046	1,838	1,852	430	0.83	0.48	5.95	4.59 ± 0.11	0.98 ± 0.01	712,341	129,927

The number of mzRT features containing the CHO, CHON, and CHOS formulas, the magnitude-averaged ratios of H/C and O/C and the double bond equivalent (DBE) values were determined (Koch et al., 2005; Sleighter and Hatcher, 2008) and used to determine compound saturation, oxygen saturation, double bond equivalent and the degree of unsaturation or ring closure for the carbon backbone, respectively. Indices including Shannon Weiner and Simpson with the total number of ASV reads for each of the samples sampled using the Omnipore filters (1 L volume). ^1^S/S Day 0 and D/S Day 0 = Surface sample and S/D Day 0 = Deep sample because of time and water budget restraints. ^2^D/S Day 0 = Deep sample because of time and water budget restraints.

### Nutrient concentrations in the experimental treatments

**Figure 5:**
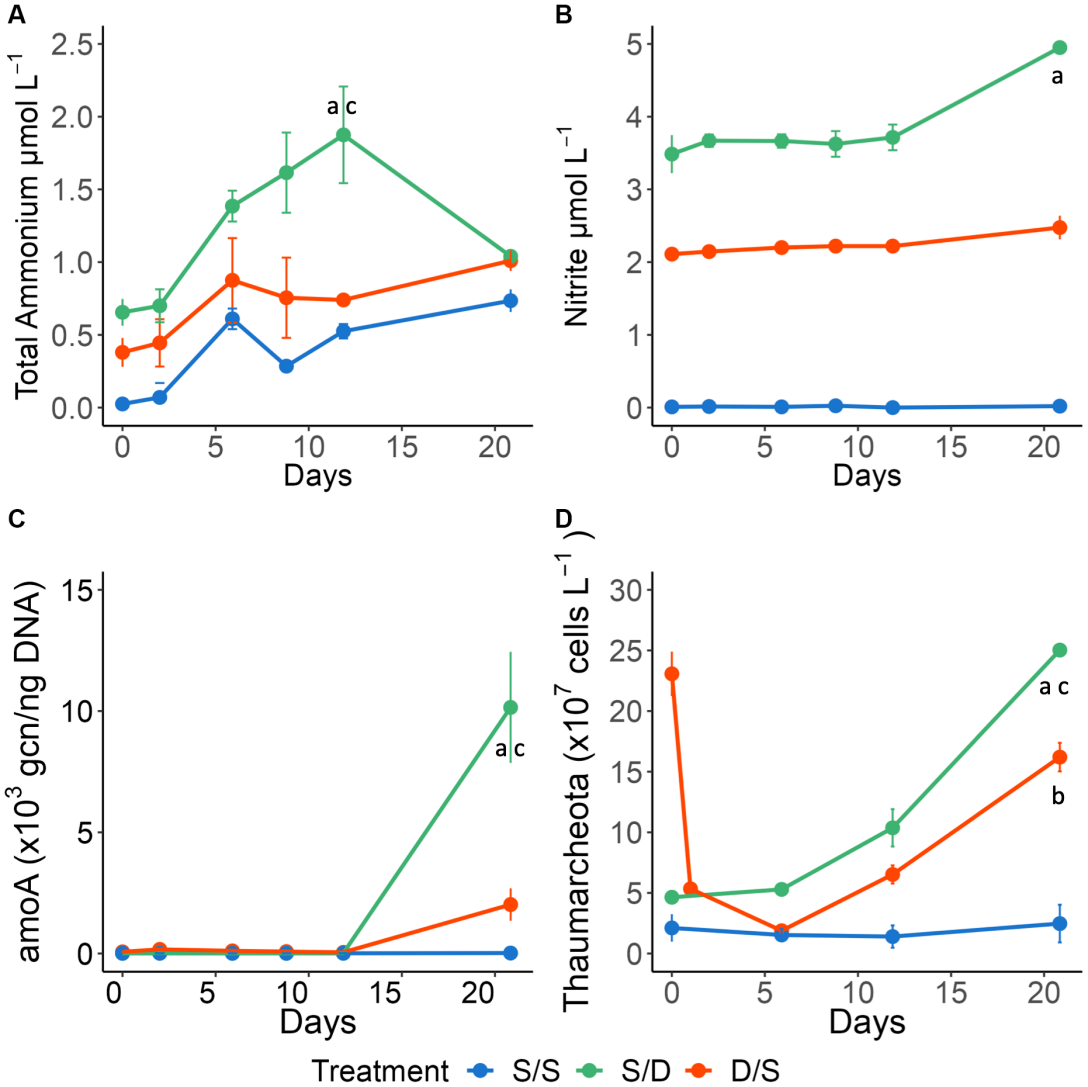
Line graphs showing **(A)** nitrite concentrations (μmol L^−^) and **(B)** total ammonium concentrations (μmol L^−1^); **(C)** the cbbL gene copy number normalized to DNA concentrations (ng) and **(D)** Thaumarcheota cell abundance (×10^7^ cells L^−1^) for all treatments over 21 days. Significant differences between treatments on specific timepoints are indicated by “a” for the S/S vs. S/D treatments, “b” for the S/S vs. D/S treatments and “c” for the S/D vs. D/S treatments.

Total ammonium, nitrite, and silicate concentrations were distinct for each treatment (Figures 5A,B; Supplementary Figure S1D), while nitrate and phosphate concentrations were consistently low and more similar between treatments (Supplementary Figures S1B,D). There were no significant differences between replicate bottles with *p* values ranging from 0.133 to 0.443 (*n* = 18). All nutrient concentrations except nitrate were highest in the S/D treatment (Figure 5; Supplementary Figure S1). The total ammonium concentrations increased twice as much in the S/D treatment compared to the other two treatments and then decreased after 12 days while the other two treatments increased (Figure 5A). Total ammonium concentrations were significantly different in the S/D treatment compared to the control (ANOVA and Tukey HSD, *p* < 0.01). Nitrite concentrations in the S/D treatment and D/S treatment were 3.48 μmol L^−1^ higher and 2.10 μmol L^−1^ higher than the control at the initial time point, respectively (Figure 5B). Nitrite concentrations remained constant in the S/S control over 21 days while increasing by 33% in the S/D treatment and 11% in the D/S between days 12 and 21. Nitrite concentrations differed significantly between all treatments (ANOVA and Tukey HSD *p* < 0.01). Silicate concentrations in the S/D treatment were 2.62 μmol L^−1^ higher than the control, while the silicate concentrations in the D/S treatment were 1.85 μmol L^−1^ higher than the control at the initial timepoint, remaining at these concentrations throughout the 21 days (Supplementary Figure S1D). Silicate concentrations were significantly different between all treatments (ANOVA and Tukey HSD *p* < 0.01).

### Change of prokaryotic lineages over the experiment

*Synechococcu*s cell abundance was determined using microscopy (Figure 3F). At the start of the experiment, there were more *Synechococcus* cells at the surface when compared to the deep (Figures 1H, 3F). As expected in the dark incubation, *Synechococcu*s cell abundance decreased by a magnitude in the S/S and S/D treatments over 4 days, with cell reduction continuing over the 21-day experiment. However, surprisingly, in the D/S treatment, there was an increase in *Synechococcu*s cells after 1.5 days, corresponding to an increase in the *cbbL* gcn on day 2 (Figures 3D,E), followed by rapid cell reduction after day 3 with *Synechococcu*s cell numbers continuing to decrease through the experiment. Since *Synechococcu*s cell abundances generally decreased in all treatments throughout the experiment, there were no significant differences seen between the treatments over all timepoints (ANOVA and Tukey HSD *p >* 0.920).

SAR202 cell abundance was determined using FISH (Figure 3E). There were 7 times more SAR202 cells in the ambient deep community when compared to the ambient surface community at the initial time point. SAR202 numbers remained low in the control treatment throughout the experiment while decreasing in the D/S treatment over 12 days before increasing again by day 21 at a rate of 5.1 × 10^6^ cells L^−1^ day^−1^. In the S/D treatment, SAR202 continuously increased in abundance by 1.1 ± 0.1 × 10^8^ cells L^−1^ over 21 days, with production rates of 3.1 × 10^6^ cells L^−1^ day^−1^ for the first 12 days and increasing to 7.6 × 10^6^ cells L^−1^ day^−1^ between days 12 and 21. SAR202 cell abundances were significantly different between the two treatments (S/D and D/S) when compared to the control (S/S), meeting the 95% confidence limit (ANOVA and Tukey HSD *p* = 0.035 for both comparisons).

Thaumarcheota cell abundance was determined using CARD-FISH and included members of the ammonia-oxidizing *Nitrospumillales*. At the start of the experiment, there were 5 times as many Thaumarcheota cells in the ambient deep community, when compared to the ambient surface community. Thaumarcheota cell abundance remained low in the S/S treatment throughout the experiment but increased in the S/D treatment, reaching a high of 2.5 ± 0.3 × 10^8^ cells L^−1^ by day 21, ~10 times that of the control treatment (Figure 5D). Thaumarcheota cells initially died off in the D/S treatment as the deep community mixed with surface DOM, low total ammonium levels, and oxic conditions (Figures 1, 5). However, as total ammonium levels increased by Day 6, Thaumarcheota cell abundance increased, reaching 1.6 ± 0.1 × 10^8^ cells L^−1^ by day 21, ~6 times that of the control treatment. While there were differences in Thaumarcheota cell abundances on day 21, there were no significant differences seen between the treatments over all timepoints (ANOVA and Tukey HSD *p* > 0.01).

### Change of specific genes over the experiment

To assess potential chemoautotrophy at the later stages in the S/D and D/S treatments as indicated from DOM data, the DNA sampled using both the Omnipore and polycarbonate filters was used to determine the gene copy number (gcn) for the *cbbL* gene and *amoA* gene using quantitative qPCR. The *cbbL* gene occurred within Devil’s Hole (Figure 1), where the oxic community consisted of the autotrophic *Synechococcus,* while the suboxic community contained SAR324, a chemolithoautotroph capable of fixing carbon using the RuBisCo pathway. The *amoA* gene also occurred within Devil’s Hole (Figure 1), where Thaumarcheota, an AOA, is abundant within the oxycline. The normalized *cbbL* gcn was high at the start of the experiment in the S/D and control (S/S) treatments where surface autotrophs were abundant (Figure 3D). The S/S control and S/D treatments showed a slight increase of ~550 and ~2,650 gcn, 650 gcn respectively, in the normalized *cbbL* gcn on day 2 and then decreased throughout the three-week experiment. The D/S treatment showed that the normalized *cbbL* gcn increased by ~18,250 gcn250 gcn between days 0 and 2 before decreasing to a minimum by day 21. The normalized *amoA* gene was present at <15 gcn within the surface seawater and at <75 gcn within suboxic water at the start of the experiment (Figure 5C). The control treatments remained low over time at <50 gcn50gcn. Both the S/D and D/S treatments showed increased *amoA* gene copies from day 12 to day 21, with an increase of ~10,130 gcn in the S/D treatment and ~1,970 gcn970gcn in the D/S treatment between days 12 and day 21.

### The prokaryotic community in the experimental treatments

When 1 L of sample was filtered using 0.2 μm Omnipore 47 mm filters, there was an average of 96,466 ± 23,326 ASV reads with a 24% coefficient of variance for 20 samples (Table 2). When ~200 mL of the sample was filtered using 0.2 μm polycarbonate 25 mm filters, there was an average of 44,408 ± 17,405 ASV reads with a 39% coefficient of variance for 30 samples (Supplementary Table S4). The DNA sampled from the polycarbonate filters had lower reads and higher error than the DNA sampled from the Omnipore filters. However, both sets of filters had similar diversity indices (Table 2; Supplementary Table S4).

The diversity of the prokaryotic community based on ASVs was determined using Shannon Weiner and Simpson indices. The deep community was lower in ASV diversity than the surface community (Table 2). The prokaryotic diversity in the S/S treatment was relatively stable over 21 days, while the prokaryotic diversity in the S/D treatment decreased over, time: reaching a minimum by day 21. The prokaryotic diversity in D/S treatment increased over 6 days to similar values to the control and then decreased over the remainder of the incubation.

The initial deep prokaryotic community was dominated by *Chlorabiales,* making up 75% of the ASVs in this sample (Figure 4C), but became unresolvable by day 2 in the D/S treatment. The microbial assemblages in the surface communities of the S/S and S/D treatments responded similarly, with a small portion of archaea seen in the deep filtrate S/D treatment, suggesting some cells passed through the filters when generating the 0.2 μm deep filtrate. By day 2, all the treatments had similar community structure with a combination of *Synechococcus*, SAR11, *Rhodospirillales, Rhodobacterales, Pseudomonadales*, and *Flavobacteriales* making up between 60 and 70% of total ASVs (Figure 4C). The control treatment community remained similar until day 21 when *Actinomarinales*, *Chitinophagales,* and *Planctomycetales* increased, while *Pseudomonadales* and *Flavobacteriales* decreased (Figure 4C). The community changed earlier by day 12 in the S/D treatment with an increase in SAR202, *Nitrospumilales*, Marine Group II, *Actinomarinales,* and *Planctomycetales,* while *Pseudomonadales* and *Flavobacteriales* decreased (Figure 4C). By day 21, the community continued to change, with an increasing contribution of SAR202, *Nitrospumilales*, and *Actinomarinales,* making up 43% of the total ASVs. The community also changed between day 9 and day 12 in the D/S treatment, with an increase of *Planctomycetales* coinciding with an increase in DOC concentrations (Figure 2). The community continued to change between days 12 and 21, with an increase in SAR202, *Nitrospumilales*, *Chitinophagales*, *Actinomarinales*, and *Flavobacteriales* that combined make up 47% of the total ASVs.

## Discussion

### The accumulation and chemodiversity of suboxic DOM

Interest in the expansion of oxygen minimum zones has increased (Stramma et al., 2008; Schmidtko et al., 2017; Stramma and Schmidtko, 2019), with DOM degradation in anoxic waters becoming a popular topic of recent research (Pantoja et al., 2009; Maßmig et al., 2019; Lau and Del Giorgio, 2020; Engel et al., 2022). The accumulation of organic carbon in anoxic environments has previously been explained by thermodynamic limitations of DOM degradation (Lee, 1992; Lau and Del Giorgio, 2020) where otherwise bioavailable DOM may accumulate as a result of hypoxia. According to the hypoxic barrier hypothesis complete DOM oxidation is inhibited at low oxygen concentrations (Giovannoni et al., 2021). This hypothesis suggests that microbial metabolism of certain types of complex DOM is dependent upon the activity of oxygenase enzymes that require free oxygen levels >25 μmol L^−1^ to initiate the enzyme. If oxygen levels are too low to meet the requirement of catabolic oxygenase enzymes, DOM degradation will not occur and DOM will accumulate. DOM accumulation can also be partially explained by the production of DOM via chemoautotrophy (Bayer et al., 2022), which is widespread in anoxic waters (Keil et al., 2016). DOM accumulated within the suboxic layer of Devil’s Hole with DOC concentrations 21 μmol C L^−1^ higher at 24 m compared to surface values at the time of experimental set-up (Figure 1).

As a labile component in the DOC pool, TDAA increased with depth at Devil’s Hole with concentrations 425 nM higher at 24 m than at the surface at the time of experimental set-up (Supplementary Table S3), suggesting its accumulation of less diagenetically altered DOM in the suboxic water. This accumulation might be related to reduced DOM degradation in the suboxic water, as seen in a recent study from the Peruvian upwelling (Maßmig and Engel, 2021). This study showed that carbohydrate concentrations declined in suboxic waters, while amino acids only decreased slightly with compositional changes indicative of bacterial peptide degradation (Maßmig and Engel, 2021). Alternatively, the accumulation of amino acids in the deep could also be explained by labile DOM production, with the amino acids alanine and threonine increasing in suboxic waters, suggesting that there is dissolved organic nitrogen production or release under suboxic conditions (Maßmig and Engel, 2021). In general, the change in the molar fraction of all amino acids was similar over depth within the Devil’s Hole OMZ in August (Supplementary Figure S2; Figure 4B). However, when looking at individual amino acid concentrations, alanine and threonine concentrations increased with depth, with deep suboxic concentrations 40 and 20 nM higher than the surface, respectively (Supplementary Table S3). The deep suboxic DOM also had elevated glycine levels when compared to the other amino acids. In fact, the increase of glycine from surface to deep made up 27.6% of the difference between the surface and deep TDAA concentrations at the time of experimental set-up (Supplementary Table S3). Glycine is considered more resistant to degradation and can accumulate in the cell walls of prokaryotes attached to sinking particles eventually making its way into the DOM pool at depth (Hecky et al., 1973; Dauwe et al., 1999). Glutamic acid is concentrated in the cell plasma and as a result tends to be depleted during degradation (Dauwe et al., 1999). This amino acid, while also higher in the deep DOM from the Devil’s Hole OMZ, only made up 7.5% of the difference between surface and deep TDAA concentration during experimental set-up (Supplementary Table S3).

During the experimental set-up, the degradation index was 2.29 at the surface and 2.47 in the deep (24 m), suggesting that there was little difference in DOM diagenetic status between the two water depths with actual TDAA concentrations higher at depth (Supplementary Table S3). However, with the deepening of the OMZ prior to the physical overturn in October, the amino acid concentrations, including alanine, threonine, glycine, and glutamic acid, increased in the deep suboxic waters at 24.5 m (Supplementary Table S3). The molar percentage of glycine decreased from 29% at the surface to 17% at depth, while the molar percentage of glutamic acid increased from 8% at the surface to 14% at depth (Supplementary Figure S2). All these factors, along with an increase in the degradation index from 0.66 at 23 m to 3.48 at 24.5 m in September (Supplementary Table S3), indicate a source of fresh dissolved organic nitrogen production. The source of this fresh production could include the solubilization of sinking POM, chemolithoautotrophy (Bayer et al., 2022), anoxygenic photoautotrophy, or release from the sediments (Kaiser and Benner, 2009). Coastal marine sediments can be hot spots of microbial dark carbon fixation (Dyksma et al., 2016). In fact, DOM released from sediment metabolic processes can serve as an important source of bioavailable DOM for microbial communities at the sediment–water interface (Loginova et al., 2020). However, since the Devil’s Hole water column is illuminated (Parsons et al., 2015), anoxygenic photoautotrophy by low-light adapted *Chlorobiales* (Overmann et al., 1992) could also be responsible for this fresh production of DOM.

Oxygen depletion can increase chemodiversity, along with the accumulation of relatively high-molecular-weight compounds enriched with carboxyl-group structures, as shown in a seasonally stratified fjord (Chen et al., 2022). The number of mzRT features increased slightly in suboxic waters, with those defined by DOM compound classes increasing by 288 mzRT features, showing increased chemodiversity in the suboxic waters of Devil’s Hole (Table 2). The DBE_w_ value was slightly elevated in the deep suboxic waters compared to the surface, suggesting increased aromatic compounds under decreased oxygen (Table 2). When comparing the mzRT features defined by DOM compound classes, surface waters contained more black carbon compounds, while deep suboxic waters contained more sugar compounds (Figure 4B). Black carbon compounds are polycyclic aromatics that are often associated with sedimentary organic carbon deposited into the ocean from river deposition (Masiello and Druffel, 1998) and make up a large proportion of total organic carbon in subtropical systems (Chew and Gallagher, 2018). Chemical composition shifts in suboxic waters appear to be influenced by a combination of decreasing oxygen (Lønborg et al., 2020; Chen et al., 2022), chemolithoautotropy (Loginova et al., 2020), DOM release (Loginova et al., 2020), and DOM preservation, with increased chemical complexity in hypoxic sediments (Jessen et al., 2017; Lau and Del Giorgio, 2020). However, the relative contribution of any of these processes remains unresolved.

### The prokaryotic community within the suboxic zone of Devil’s Hole

**Figure 6:**
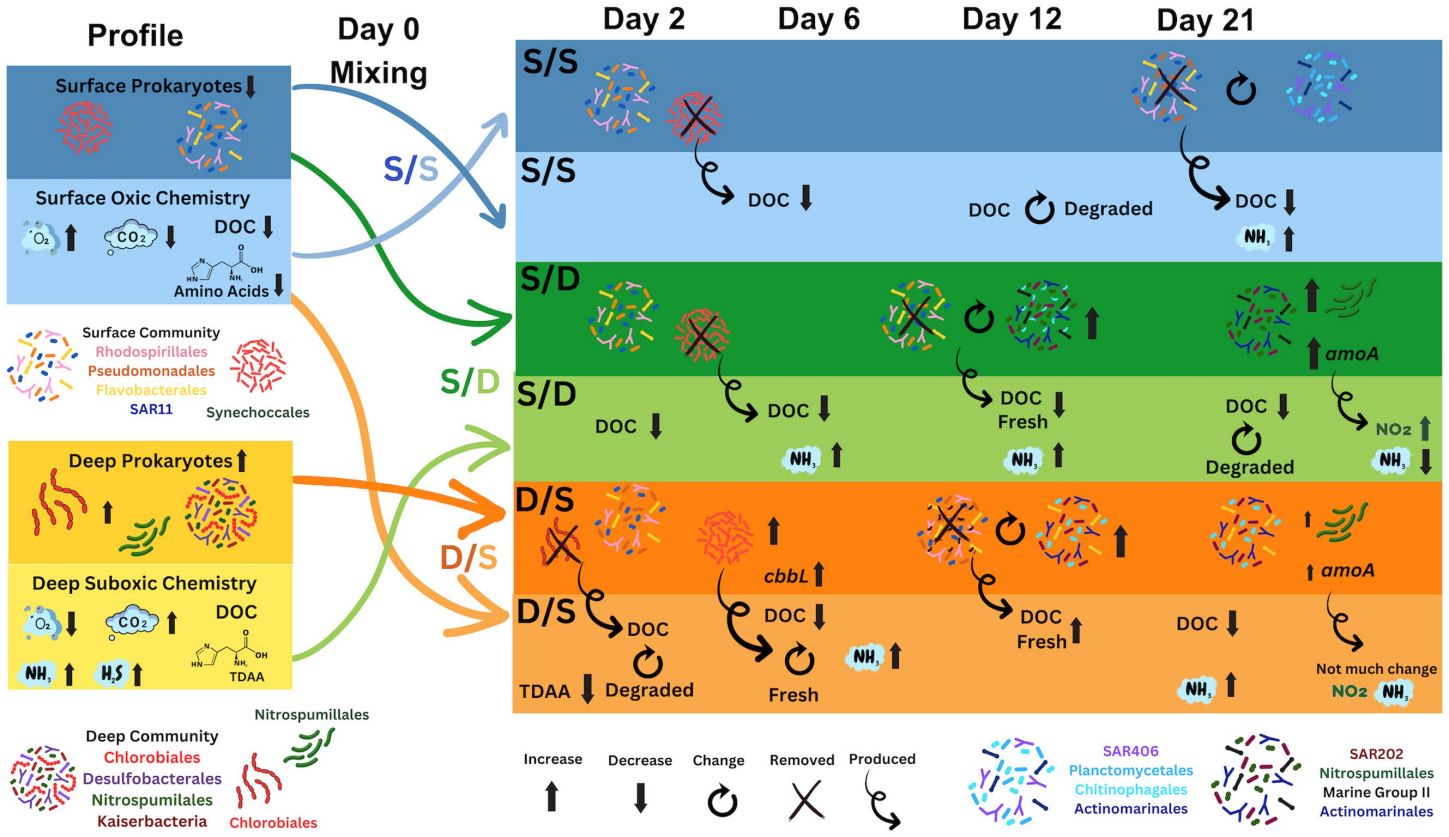
Diagram showing the prokaryotic community change in association with the chemistry changes during the simulated overturn experiment. Prokaryotic community is color coded in the legend and the chemistry includes ammonia (NH_3_), carbon dioxide (CO_2_), DOC, hydrogen sulfide (H_2_S), oxygen (O_2_), and nitrite (NO_2_). DOC quality (fresh and degraded) is determined using the amino acid degradation index. Gene copy number for the cbbL (carbon fixation) and amoA (nitrification) gene is also included.

The microbial communities within coastal OMZs are similar to those in open ocean OMZs (Wright et al., 2012). A previous study of the Devil’s Hole OMZ found that the abundance of *Chlorobiaceae,* a group of anoxygenic photolithoautotrophic green sulfur bacteria (GSB), increased with depth dominating the suboxic community with 59% of total prokaryotic cells (Parsons et al., 2015). In this study, ~75% of ASVs from the suboxic zone were from the family *Chlorobiales* and genus *Chlorobiaceae* at the time of experimental set-up (Figure 4C). The GSB is known to have two to three copies of the 16S ribosome (Stoddard et al., 2015), explaining the overabundance of this genus in the sequencing data. This GSB species mainly produce a monocyclic aromatic carotenoid, chlorobactene (Takaichi et al., 1997; Canniffe et al., 2018), a small molecule which may not have been captured on the PPL columns in our metabolite data (Dittmar et al., 2008; Johnson et al., 2017). In addition to ASVs belonging to the family *Chlorobiales*, other unique prokaryotic members of the suboxic community not present in the surface (summarized in Figure 6) include copiotrophic bacteria such as SAR406, *Floavobacteriales, Rhodospirialles,* and *Rhodobacterales* along with the archaea, *Euryarcheota* from the family Marine group II and *Thaumarchaeota* from the family *Nitrosopumilales*, and the bacteria *Desulfobacterales,* a known sulfate reducer present along with members of *Candidatus Kaiserbacteria* that belong to the Candidate Phyla Radiation (CPR) bacterial group with diverse metabolisms (Brown et al., 2015).

### The surface prokaryotic community response to deep DOM during exponential phase growth

The prokaryotic response was distinct for the S/D treatments (Figure 2A), with the highest specific growth rate and the highest prokaryotic growth during exponential phase (Figure 2B). The S/D treatment had the highest BGE of all the treatments (Table 1), suggesting that it assimilated the most carbon, converting it into biomass. BGE estimates in the Red Sea ranged from 2 to 13% depending on the time of year, with summer having the highest growth efficiencies (Silva et al., 2018). The authors suggested that temperature drove the higher BGE values in their study which had similar sea surface temperatures (33°C) when compared to our study (30°C). Since BGE can also be restrained by the quality of the available substrate (Vallino et al., 1996; Farjalla et al., 2009), this high BGE of 17.9% in the S/D treatment suggests that there is enough substrate during exponential phase (Table 1). A mixture of fresh labile and accumulated refractory DOM increased bacterial growth in bacterial batch cultures where nitrogen and phosphorus were added (Farjalla et al., 2009), resulting in BGE of >10%. Since nutrients were not limiting in the present study (Figure 5; Supplementary Figure S1) and the DOM pool consisted of a mixture of labile and refractory DOM (Figures 4A,B), the high BGE is not surprising.

In the S/D treatment, both DOC and TDAA were removed at greater rates than the control over the first 6 days (Figures 2, 3) with little drawdown of nutrients (Figure 5; Supplementary Figure S1). As a result, neither macronutrients nor the concentration or quality of DOM that accumulated under anoxic conditions appeared to be limiting to heterotrophic removal once the water was reoxygenated and mixed with surface microbial assemblages. A study from the Baltic Sea suggested that the bacterial turnover of organic matter was limited by nutrients (total ammonium, phosphate, nitrate) under both oxic and suboxic conditions (Maßmig et al., 2019). Another study in coastal Chilean OMZ showed that the quality of the organic matter rather than oxygen concentration controlled the heterotrophic degradation of DOM (Pantoja et al., 2009). In the present study, compositional changes in amino acids were observed with decreasing molar proportions of glutamic acid, aspartic acid, serine, and leucine over the first 6 days (Figure 4A). These more bioavailable amino acids are considered more labile as inferred from the DI index (Dauwe et al., 1999). Their preferential removal in the S/D treatment is consistent with previous work conducted in the Peruvian OMZ that demonstrated comprehensive and preferential remineralization of these amino acids by microbes within the upper oxycline (Loginova et al., 2019).

Within the first 2 days of the present study, 37% of DOC was removed in the S/D treatment, while between days 2 and 6, DOC removal reduced to only 5% of total DOC removal (Figure 2) with an increase in proportions of *Synechococcales* and *Flavobacteriales* (Figure 4C). Members of the *Flavobacteriales* clade are known to degrade both low and high molecular weight DOM compounds (Cottrell and Kirchman, 2000) but some ASVs specialize in polymer degradation (Fernandez-Gomez et al., 2013). In the Baltic Sea, seawater culture regrowth experiments enriched with labile DOM compounds showed that *Flavobacteriales* dominated after polypeptide amendments (Pontiller et al., 2020). In this experiment, the prokaryotic community shifted with *Synechococcales* disappearing and *Flavobacteriales* persisting after 6 days along with an increased in peptide compounds (Figures 4B,C) suggesting that this family could be responsible for breaking down polypeptides to peptides within the first 6 days of the experiment.

### Chemolithoautotrophy as the surface prokaryotic community responds to deep DOM

After day 6, while DOC concentrations decreased further (Figure 2C), TDAA concentrations increased by 14 nM, and the degradation index increased between days 6 and 12 in the S/D treatment (Figures 3A,B), suggesting that the quality of DOM shifted to a less diagenetically altered status. This change is further corroborated by the decreasing molar contribution of alanine and glycine, two amino acids that are more resistant to degradation, when compared to the control during the same time frame (Figure 4A). The labile DOM might come from new production by chemolithoautotrophy during the incubations, as *Nitrosopumilus* strains have been shown to fix carbon (Könneke et al., 2014; Liu et al., 2021), and produce DOM consisting of labile amino acids such as alanine, leucine, and serine (Bayer et al., 2019). In this study, *Thaumarcheaota* cell abundances increased from days 6 to 21 in the S/D treatment (Figure 5D). and *Nitrosopumilales ASVs* increased to 18% of total prokaryotic cells in the S/D treatment by day 21 at the same time as the shift to less diagenetically altered DOM (Figures 3B, 5D, 4C; Supplementary Figure S5).

*Thaumarcheaota,* including the family *Nitrosopumilales,* are chemoautotrophic ammonia-oxidizers that play an essential role in nitrogen and carbon cycling (Könneke et al., 2005; Beman et al., 2008; Santoro et al., 2015; Garritano et al., 2022) and can be found in the oxycline of OMZs (Lin et al., 2006; Zaikova et al., 2010; Belmar et al., 2011; Wright et al., 2012) including the oxycline of Devil’s Hole (Parsons et al., 2015). An increase in Thaumarcheota also occurred in the coastal China Sea where Thaumarcheota cells transform ammonia into nitrite via chemoautotrophic ammonia oxidation (Xie et al., 2020; Wang et al., 2022). Since the *amoA* normalized gcn increased over 124 times within the S/D treatment between days 12 and 21 (Figure 5D), ammonia oxidation could be occurring in this treatment. The occurrence of this pathway was confirmed by a doubling of nitrite concentrations and a coincident decrease in total ammonium concentrations (by half) in the S/D treatment between days 12 and 21, when compared to the control treatment (Figure 5).

### Deep DOM degradation and nutrient cycling by specific microbial lineages

Despite the possible fresh DOM production in the later stages of S/D treatment, DOC concentration continued to decrease with recalcitrant components of DOM apparently being removed or transformed, as shown in the metabolite data with a slight decreasing percentage of CRAM contribution over time (Figure 4B). SAR202, a chloroflexi, increased from 3 to 14% of the total prokaryotic ASVs and increased from 3 to 13% of total prokaryotic cells in the S/D treatment between day 12 and 21 (Figures 3E, 4C). At the same time, SAR202 cells also increased by 6.9 × 10^8^ cells L^−1^ while DOC concentrations decreased by an additional 30% (Figures 2C, 3E). Genomic and metagenomic studies have postulated that SAR202 cells have the enzymatic repertoire capable of oxidizing some forms of recalcitrant DOM (Landry et al., 2017; Mehrshad et al., 2018; Saw et al., 2020), which may be occurring in our study (Figure 6).

Along with SAR202, *Planctomycetales* ASVs responded to the deep DOM increasing by day 12, along with the *Euryarcheota* Marine Group II and *Actinomarinales* in the S/D treatment (Figure 4C). Marine Group II continued to respond to the S/D experimental conditions, contributing to 13% of the total prokaryotic ASVs by day 21 (Figures 4C,D). *Planctomycetes, Actinomarinales, Flavobacteriales*, and Marine Group II were present on the S/D treatment on day 21 (Figures 4C,D), coinciding with a decrease in proteins and peptides in the metabolites data (Figure 4B), suggesting they may have a role in protein cycling. Other studies showed that members of *Planctomycetacia, Flavobacteriales,* and Marine Group II actively degraded *Synechococcus*-derived organic matter in the China Sea, suggesting their important role in the degradation of labile DOM (Xie et al., 2020; Wang et al., 2022). These groups were also involved in the cycling of dissolved proteins and dissolved organic nitrogen within the California Current (Orsi et al., 2016). In addition, *Planctomycetales* may be critical to changing DOM quality (Tadonleke, 2007) and involved with DOM degradation based on experiments using natural DOM additions in the estuarine Baltic Sea (Traving et al., 2017), suggesting its role in the degradation of the deep formerly suboxic DOM at Devil’s Hole.

### The initial response of deep prokaryotes to surface DOM

Since the deep community is adapted to low oxygen conditions, introducing of oxygen to the community would simulate convective overturn that results in both a redistribution of cells and a community shift back to surface communities (Parsons et al., 2015). In fact, total prokaryotes from the suboxic depths had an initial 25% cell die-off when mixed with oxygenated surface DOM (Figure 2A). But afterwards, the D/S treatment has a higher specific growth rate and prokaryotic growth during exponential phase when compared to the control (Table 1; Figure 2B). The BGE in the D/S treatment was also higher than the control, suggesting that the quality of the available substrate was different from the control (Vallino et al., 1996; Farjalla et al., 2009). Since the D/S treatment had 70% of the DOM pool from the surface and 30% from the deep, this suggests that the deep DOM pool provided enough nutrients (Figure 5; Supplementary Figure S1) and the mixture of labile and refractory DOM (Figures 4A,B) that can enhance prokaryotic growth (Farjalla et al., 2009). While DOC concentrations were similar to the control at the start of the experiment, nutrients and TDAA were higher than the control showing the enrichment of these compounds in the unfiltered deep water. Thus, the response of deep prokaryotes to the surface DOM may just be a result of the deep DOM in the whole unfiltered prokaryotic community portion of the D/S treatment. TDAA were removed rapidly in the D/S treatments within the first 2 days, almost triple the removal seen in the control (Figure 3A). By day 2, the degradation index decreased to 0.55 ± 0.73 in the D/S treatment (Figure 3B) suggesting that the surface DOM was rapidly degraded by the changing prokaryotic community. The dominant amino acids, alanine, aspartic acid, glutamic acid, leucine and serine (Figure 4A) all decreased rapidly in the D/S treatment by day 2 (Supplementary Figure S3). Preferential uptake of glutamic acid, serine and leucine by microbes has occurred in the upper oxycline of the Peruvian OMZ (Loginova et al., 2019), this rapid decrease of these amino acids is not surprising given that the prokaryotic community is transitioning early in the incubation.

### The deep prokaryotic community response to surface DOM

*Chlorobiales* initially dominated the deep prokaryotic community in the D/S treatment (Figure 4C). Dividing by the three copies of the 16S ribosome found in *Chlorobiales* would lower the relative contribution of this family from 75% of total ASVs to 25% of total ASVs at the beginning of incubation, more similar to previous studies (Parsons et al., 2015). *Chlorobiales* are anoxygenic photoautotrophic GSBs that require reduced sulfur compounds as electron donors (Muyzer and Stams, 2008). During sampling, suboxic seawater was reoxygenated, and gasses that built up in the sediment–water interface, such as ammonia, hydrogen sulfide, and methane, were released into the atmosphere (Thorstenson and Mackenzie, 1974). Since *Chlorobiales* requires anaerobic conditions and reduced sulfur compounds (Muyzer and Stams, 2008), this family of bacteria would not survive for long in oxygenated conditions and become reduced to below the level of detection in the ASV data over the first 2 days (Figure 4C).

The prokaryotic community continued to change from days 0 to 2, resulting in a community more similar to the other two treatments with retention of the Archaea MGII and an increasing percentage of *Flavobacteriales* in the D/S treatment (Figures 4C, 6), consistently indicating their role in labile DOM degradation. Different from other two treatments, the contribution of *Synechococcales* in the D/S treatment increased in the first few days for both the sequencing data (Figure 4C) and cell counts via microscopy (Figure 3F). This corresponds with the 14-fold increase in *cbbL* gene abundance at day 2, suggesting autotrophy by *Synechococcus* since low oxygen concentrations can shift RuBisCO transcripts from photoautotrophy to chemoautotrophy in *Synechococcus* cultures (Han et al., 2022). The specific *Synechococcales* found in the D/S treatment on day 2 were CC9902 and *Cyanobium* PCC-6307. Recent studies in the Black Sea have shown that viable *Synechococcus* strains, including *Cyanobium,* have adapted to dark conditions (Callieri et al., 2019, 2022).

Between day 2 and day 6, TDAA concentrations, TDAA yield, and the degradation index increased in the D/S treatment (Figure 3B). This suggests an influx of less diagenetically altered DOM that might come from intracellular DOM release after the death of *Chlorobiales* and *Synechococcales* cells that disappear in the D/S treatment during this period (Figures 3F, 4C). Leucine concentrations increased five times during this time period (Supplementary Figure S3E), which further supported the explanation of fresh DOM from the dead cells, as leucine serves as an essential component for prokaryotic protein synthesis (Simon and Azam, 1989). By day 6, members of *Planctomycetales* began to increase in ASV abundance, and *Flavobacteriales* ASVs increased from day 0 to day 6 and made up 14% of the total ASVs (Figure 4C; Supplementary Figure S5), again showing their role in labile DOM degradation.

### Changes in DOM quality and concentrations as the prokaryotic community changes in the D/S treatment

Between days 9 and 12, DOC concentration increased in the D/S treatment by 5 μmol C L^−1^ (Figures 2C, 6). TDAA yield and DI at day 12 remained high, while the more resistant amino acid, glycine, decreased in its molar percentage during this time period (Figure 4A). This fresh DOM production might be attributed to a community change seen with *Planctomycetes* responding between days 9 and 12 (Figure 4C). *Planctomycetes* have been associated with DOM quality changes through degradation (Tadonleke, 2007) and the production of novel small molecules, including polyketides and non-ribosomal peptides (Wiegand et al., 2020). However, this change in DOC concentrations and quality may also involve the chemoautotrophic *Thaumarcheaota* since cell abundances increased in the D/S treatment by day 21. At the same time, the *amoA* normalized gene copy number doubled by day 21 (Figures 5C,D). Since *Nitrosopumilus* strains fix carbon efficiently (Könneke et al., 2014) and have been shown to produce labile DOM in the form of amino acids (Bayer et al., 2019), the growth of *Nitrosopumilales* from only 2% of the total prokaryotic ASVs on day 12–13% of total prokaryotic cells on day 21 in the D/S treatment could be linked to DOM production (Figures 5D, 4C).

During the later stage in the D/S treatment, the proportion of *Planctomycetales* and Marine Group II increased between day 6 and day 12, suggesting their potential role in degrading labile DOM (Figures 4C, 6; Supplementary Figure S5). Coinciding with a slight decrease in the CRAM fraction (Figure 4B), SAR202 cell abundance and the SAR202 ASV proportion to total ASVs increased by day 21 in the D/S treatment (Figures 3E, 4C). Since SAR202 cells have the genetic capacity to degrade aromatic compounds like CRAM (Landry et al., 2017; Saw et al., 2020), they might be responsible for this decrease in the CARM fraction. In addition, Ga0077536 ASVs increased from days 9 to 21 in the D/S treatment, and their genomes contain many interesting genes, including those involved in methylamine utilization and several monoxygenases responsible for aromatic, linear, and cyclic aliphatic hydrocarbon degradation (Francis et al., 2021). The presence of this gammaproteobacteria by day 21 suggests that along with SAR202, these families could slowly respond to the recalcitrant DOM present by 21 days.

## Conclusion

Devil’s Hole, Bermuda, is a natural laboratory where thermally driven stratification causes the formation of a seasonal OMZ during the summer months. During the summer months, when suboxic conditions develop, organic matter accumulates within the suboxic layer. The bioavailability of this accumulated DOC and the microbial community response to reoxygenation of suboxic waters showed that prokaryotes responded to the deep formerly suboxic DOM with high growth rates coinciding with increased DOC drawdown. The prokaryotic community changed within 2 weeks with increasing contributions of *Planctomycetales*, SAR202, and the chemoautotrophic ammonia-oxidizing, *Nitrospumillales*. In contrast, deep prokaryotes had an initial die-off before shifting to a surface-like community devoid of *Chlorobiales*, the major taxa in the suboxic zone. cbbL gene abundance increased on day 2 followed by a change in DOM quality to less diagenetically altered material by day 6. DOC concentrations increased between days 6 and 12, while the prokaryotic community changed to include *Planctomycetales*. By the end of the experiment, this treatment also increased in SAR202 and *Nitrospumillales*. With reoxygenation, the deep DOM that accumulated under suboxic conditions is bioavailable to surface prokaryotes that utilize the accumulated DOC initially before switching to a community that can both produce less diagenetically altered DOM via chemoautotrophic and degrades the more recalcitrant DOM.

## Scope

Devil’s Hole, Bermuda is a shallow inland system that experiences seasonal anoxia. Devil’s Hole represents a natural laboratory where microbial-driven processes and community succession can be studied in the illuminated water column. The stratification and the development of anoxia, along with subsequent overturn and the reoxygenation of the water column, has been studied as part of a monthly time-series. As temperature-driven stratification occurs, oxygen levels decrease to suboxic levels and DOM accumulates. Simulated overturn experiments were used to mimic mixing; thereby assessing the bioavailability of the accumulated dissolved organic carbon (DOC) to the surface microbial community. The surface prokaryotic community responded to the deep (formerly suboxic) filtrate within 6 days. The DOM quality changed to less diagenetically altered material and coincided with a community shift to prokaryotes that can both produce labile DOM via chemoautotrophy and degrade the more recalcitrant DOM. For example, nitrification occurred with an increase in *Nitrosopumilales,* chemoautotrophic ammonia oxidizing archaea (AOA) that converted ammonia to nitrite based on the ammonia monooxygenase (*amoA*) gene copy number and nutrient data. This study shows that suboxic DOM is bioavailable to surface prokaryotes that in turn, drive key processes that influence the carbon and nitrogen cycles.

## Data availability statement

The datasets presented in this study can be found in online repositories. The names of the repository/repositories and accession number(s) can be found in the article/Supplementary material.

## Author contributions

RP: Conceptualization, Data curation, Formal analysis, Investigation, Methodology, Project administration, Resources, Validation, Visualization, Writing – original draft. SL: Conceptualization, Data curation, Formal analysis, Supervision, Visualization, Writing – review & editing. KL: Data curation, Formal analysis, Visualization, Writing – review & editing. KY: Data curation, Investigation, Methodology, Writing – review & editing. CJ: Data curation, Investigation, Methodology, Writing – review & editing. LB: Data curation, Methodology, Software, Supervision, Writing – review & editing. JC: Data curation, Methodology, Writing – review & editing. KO: Data curation, Methodology, Writing – review & editing. MK: Data curation, Methodology, Writing – review & editing. RG: Data curation, Investigation, Methodology, Writing – review & editing. CC: Funding acquisition, Conceptualization, Resources, Supervision, Writing – review & editing. BT: Conceptualization, Resources, Supervision, Data curation, Methodology, Software, Writing – review & editing. NB: Funding acquisition, Project administration, Resources, Supervision, Writing – review & editing.

## Footnotes

^1^http://analab.msi.ucsb.edu/
