# Supplementary material for: Suboxic DOM is bioavailable to surface prokaryotes in a simulated overturn of an oxygen minimum zone, Devil’s Hole, Bermuda
Source: Front Microbiol. 2023 Dec 20;14:1287477. doi: 10.3389/fmicb.2023.1287477 (PMC10765504; doi:10.3389/fmicb.2023.1287477)
Supplement: Supplementary file 1 [file Data_Sheet_1.docx]

**Supplemental Methods, Figures and Tables**

***cbbL Primer Design and Specificity***

The cbbL gene was targeted using primers designed for this study (Table S1). The cbbL gene encodes for RuBisCO, the enzyme that catalyze carbon fixation via the Calvin Bensen cycle.

**Table S1**: Sequences of the primers designed for cbbL gene amplification and the resulting amplicon.

| cbbL forward | 5'-CAC GGY ATY CAC TTC CG-3' |
| --- | --- |
| cbbL Reverse | 5'-CGT GRA TAC CRC CKG AAG CRA C-3' |
| Amplicon sequence | 5’CACGGTATCCACTTCCGTGTGCTGGCCAAGATTCTGCGTCTGTCCGGTGGTGACCACCTCCACTCCGGTACCGTGGTCGGCAAGCTGGAAGGTGACCGCGAAGCGACCCTAGGCTGGATCGACCTGATGCGCGAAGACTACGTCAAAGAAGACCGCAGCCGCGGCATCTTCTTCGACCAAGACTGGGGTTCCATGCCAGGCGTGATGCCG-3’ |

cbbL gene from Chlorella sorokiniana (NC_023835.1), Chlamydomonas reinhardtii (J01399.1), Anacystis nidulans (X03220.1), Synechococcus sp (U46156.1), Hydrogenovibrio marinus (D43622.1), T.ferrooxidans (X70355.1), Pot. Thiobacillus sp (M34536.1), Thiobacillus denitrificans (L42940.1), and Hydrogenophilus thermoluteolus (D30764.1) were aligned via Clustal O algorithm (Sievers F et al., 2011) with default settings. Primers were designed to amplify an amplicon that resides between the 2420 base pair and 2629 base pair of the aligned cbbL sequence. The forward primer binds at position 2420 – 2437 and the reverse primer binds at position 2415-2437 of the aligned cbbL gene. The sequences for primers and amplicon are provided in Table S1. In Silco analysis of secondary structure (IDT - UNAFold), melting temperature (IDT – UNAFold, IDT - OligoAnalyzer) and species specificity (NCBI – primer blast) were also undertaken to ensure primer specificity (Ye et al., 2012).

***Preparation of the qPCR Standards for amoA and cbbL***

The standards for the amoA and cbbL genes were prepared using the amoA primers (Treusch et al., 2005) and the cbbL primers designed in this study (Table S1), respectively. The cbbL and amoA qPCR standard was prepared by amplifying seawater sampled from 5m and 23m at Devil’s Hole on the day of the experimental set-up on August 9^th^ 2018, respectively. This sample was chosen as the prokaryotic composition consisted of 34% cyanobacteria cells and 10% of Synechococcus ASVs. qPCR standards were created using the TOPO™ XL-2 Complete PCR Cloning Kit (Invitrogen™) following manufacturers protocol. First, a blunt end PCR product was amplified (Table 2) and analysed on a 2% agarose gel. A single PCR product was detected, and the PCR product was purified using the PureLink™ Quick Gel Extraction and PCR Purification Combo Kit (Invitrogen™) following the manufacturer's protocol.

The PCR product was subcloned into the pCR-XL-2-TOPO™ vector by incubating the purified PCR product (4μl) with the pCR-XL-2-TOPO™ vector (1μl) and the salt solution (1μl) for 20 minutes at R.T. Upon incubation, One Shot™ OmniMAX™ 2 T1^R^ Chemically Competent E. coli cells (Invitrogen) were thawed on ice 5 minutes before transfection. 2μl of the cloning reaction was added to one vial of One Shot™ OmniMAX™ 2 T1^R^ Chemically Competent E. coli cells and gently flicked to mix and incubated for 30 minutes at R.T. The mixture was placed at 42°C for 30 seconds before being returned to ice for 2 minutes. 250μl of SOC Media (ThermoFisher) was added and cells placed in shaking incubator for 1 h (225rpm at 37°C). Transfected E.coli cells were spread onto LB agar plates (containing ampicillin (100μg/ml) and 1mM IPTG) and incubated overnight at 37°C. Ampicilin resistant transformants were analysed via colony PCR. Briefly, a picked colony was taken and PCR performed using a mastermix (25μl of Platinum ^TM^ SuperFi ^TM^ Green PCR master mix, 23μl of nuclease free water and 10mM of forward and reverse PCR primers for the gene of interest (Table S2).

**Table S2**: Thermal conditions for amoA and cbbL PCR amplification.

|  | cbbl | | | amoA | | |
| --- | --- | --- | --- | --- | --- | --- |
| Process | Temperature | Time | Cycles | Temperature | Time | Cycles |
| dUTP carry over removal | 50°C | 1 min | 1 | 50°C | 2 min | 1 |
| Initial Denaturation | 95°C | 10 min | 1 | 95°C | 2 min | 1 |
| Denaturation | 95°C | 30 sec | 40 | 95°C | 15 sec | 40 |
| Annealing and Extension | 60°C | 1 min |  | 60°C | 1 min |  |

Amplification conditions for colony PCR included: initial denaturation and bacterial lysis for 10 minutes at 94°C, followed by 35 cycles of: 98°C for 10 sec, 60°C for 10 seconds and 72°C for 30 seconds. The PCR product was then analysed on a 2% agarose gel. A single band was detected, and subsequently, another single colony was picked incubated overnight in a shaking incubator (225rpm at 37°C). The plasmid was purified using GeneJET Plasmid Miniprep Kit (Thermo Scientific™) following the manufacturer's instruction. The purified plasmid was used as standard, and the sequence was confirmed by amplicon sequencing. Amplicon sequencing was performed at UC Davis using the ABI 3730 Capillary Electrophoresis Genetic Analyzer. Purified products were quantified using a Qubit fluorometer and the cbbL gene abundance were subsequently calculated from the concentration of the purified DNA and the size fragment. Ten-fold serial dilutions ranging from 10^6^ to 10^1^ gene copies of the corresponding standard were used in duplicate per q-PCR reaction to generate an external quantification standard.

**Table S3:** Significance values of each parameter displayed in the figures determined between the three treatments (S/S vs S/D, S/S vs D/S and S/D vs D/S) using analysis of variance (ANOVA) and Tukey’s Honest Significant Difference test. **Significance (*p*) is indicated in bold.** The sample number (N) and referenced figures are also included.

|  | **Treatment** | **S/S vs S/D** | **S/S vs D/S** | **S/D vs D/S** | **Sample Number** | **Figure** |
| --- | --- | --- | --- | --- | --- | --- |
| **Parameter** | **Units** | **a** | **b** | **c** | **N** |  |
| Prokaryote | x10^8^ cells L^-1^ | **0.0000** | **0.0000** | **0.0107** | 28 | 2 |
| DOC | µmol L^-1^ | 0.9969 | 0.9912 | 0.9986 | 12 | 2 |
| TDAA C | nmol L^-1^ | 0.8104 | 0.9130 | 0.9752 | 8 | 3 |
| DI |  | 0.2199 | 0.5343 | 0.7997 | 8 | 3 |
| TDAA Yield | % | 0.8209 | 0.9090 | 0.9815 | 8 | 3 |
| cbbL | gcn ng^-1^ DNA | 1.0000 | 0.5571 | 0.5518 | 6 | 3 |
| SAR202 | x10^7^ cells L^-1^ | **0.0096** | **0.0004** | 0.3933 | 8 | 3 |
| Synechococcus | x10^7^ cells L^-1^ | 0.9988 | 0.8477 | 0.8706 | 20 | 3 |
| Ammonium | µmol L^-1^ | **0.0002** | 0.1874 | **0.0218** | 12 | 4 |
| Nitrite | µmol L^-1^ | **0.0000** | **0.0000** | **0.0000** | 12 | 4 |
| amoA | gcn ng^-1^ DNA | 0.4504 | 0.9918 | 0.5198 | 6 | 4 |
| Thaumarcheota | x10^7^ cells L^-1^ | **0.0275** | **0.0280** | 0.9600 | 8 | 4 |
| Nitrate | µmol L^-1^ | **0.0234** | **0.0000** | **0.0025** | 12 | S1 |
| Phosphate | µmol L^-1^ | 0.0017 | 0.8953 | **0.0057** | 12 | S1 |
| Silicate | µmol L^-1^ | **0.0000** | **0.0000** | **0.0000** | 12 | S1 |
| Alanine | nmol L^-1^ | 0.9450 | 0.7762 | 0.9308 | 8 | S2 |
| Aspartic Acid | nmol L^-1^ | 0.8798 | 0.9101 | 0.9974 | 8 | S2 |
| Glutamic Acid | nmol L^-1^ | 0.8212 | 0.8861 | 0.9906 | 8 | S2 |
| Glycine | nmol L^-1^ | 0.9685 | 0.9961 | 0.9433 | 8 | S2 |
| Leucine | nmol L^-1^ | 0.6690 | 0.8336 | 0.9561 | 8 | S2 |
| Serine | nmol L^-1^ | 0.9475 | 0.9999 | 0.9521 | 8 | S2 |

**Table S4:** Profile data of Total Dissolved Amino Acids (TDAA) concentration as nmol L^-1^, the Total Dissolved Amino Acids (TDAA) concentration as nmol C L^-1^, the TDAA Yield Index; the Degradation Index and the Amino Acid concentrations as nmol L^-1^ of Alanine, Threonine, Glycine and Glutamic Acid at the time of experimental set-up in August 2018 (shaded white) and just before convective overturn in September 2018 (shaded grey). Samples in bold indicate depths where oxygen levels < 20 µmol L^-1^.

| Sample Depth | Total TDAA nM | Total TDAA C nM | TDAA Yield | Degradation Index | Alanine nM | Threonine nM | Glycine nM | Glutamic Acid nM |
| --- | --- | --- | --- | --- | --- | --- | --- | --- |
|  |  |  |  |  |  |  |  |  |
| 1m | 1294.20 | 5031.39 | 5.36 | 2.29 | 154.89 | 189.24 | 273.49 | 131.79 |
| 5m | 1026.69 | 3947.72 | 4.23 | 2.12 | 114.68 | 145.15 | 235.11 | 102.60 |
| 10m | 1399.34 | 5519.55 | 5.91 | 2.69 | 161.20 | 209.47 | 292.70 | 146.50 |
| 15m | 1256.94 | 4894.36 | 5.15 | 2.24 | 151.97 | 175.85 | 262.89 | 118.00 |
| 20m | 1496.40 | 5858.31 | 6.22 | 2.42 | 170.35 | 193.03 | 325.24 | 155.13 |
| 22m | 1615.85 | 6260.21 | 5.92 | 2.24 | 193.75 | 216.33 | 353.86 | 148.18 |
| 23m | 1546.05 | 6027.57 | 5.66 | 2.36 | 169.79 | 207.27 | 354.26 | 156.48 |
| 24m | 1718.70 | 6742.42 | 5.87 | 2.47 | 197.94 | 218.89 | 388.39 | 165.06 |
| 1m | 652 | 2341 | 2.51 | 0.79 | 86.95 | 32.90 | 188.35 | 49.53 |
| 5m | 631 | 2294 | 2.55 | 1.22 | 81.22 | 41.88 | 168.95 | 58.23 |
| 10m | 718 | 2703 | 3.06 | 1.86 | 97.55 | 51.60 | 170.77 | 72.33 |
| 15m | 630 | 2297 | 2.6 | 1.17 | 83.50 | 41.53 | 161.26 | 57.87 |
| 20m | 625 | 2304 | 2.71 | 1.26 | 85.47 | 40.20 | 157.48 | 60.72 |
| 22m | 565 | 2017 | 2.34 | 0.79 | 77.86 | 40.25 | 153.27 | 52.55 |
| 23m | 560 | 2008 | 2.43 | 0.66 | 76.51 | 33.78 | 148.23 | 52.98 |
| 24.5m | 2572 | 10737 | 11.92 | 3.48 | 286.22 | 148.99 | 447.02 | 366.26 |

**Table S5**: Diversity Indices including Shannon Weiner and Simpson with the total number of ASV reads for each of the back-up samples filtered using the polycarbonate filters and 100-200mLs of volume. ASV Reads are included for both bottle replicates and range from 2837 to 105497. Error is standard deviation. The D/S Day 2 sample reads replicate one had very low reads indicated by italics and was removed from the qPCR data as the DNA concentration was too low for Qubit fluorometry.

|  | Diversity Indices | | Sequencing | |
| --- | --- | --- | --- | --- |
| Sample ID | Shannon Weiner | Simpson | #ASV Reads Rep1 | # ASV Reads Rep2 |
| S/S Day 2 | 4.33 ± 0.01 | 0.97 ± 0.00 | 47202 | 43046 |
| S/S Day 6 | 3.98 ± 0.21 | 0.96 ± 0.01 | 36711 | 71502 |
| S/S Day 9 | 4.37 ± 0.01 | 0.98 ± 0.00 | 37416 | 40231 |
| S/S Day 12 | 4.38 ± 0.03 | 0.98 ± 0.00 | 47305 | 41539 |
| S/S Day 21 | 4.23 ± 0.10 | 0.97 ± 0.00 | 57483 | 57873 |
| S/D Day 2 | 4.41 ± 0.15 | 0.97 ± 0.01 | 40564 | 36711 |
| S/D Day 6 | 3.92 ± 0.72 | 0.94 ± 0.05 | 44236 | 71523 |
| S/D Day 9 | 4.45 ± 0.13 | 0.98 ± 0.00 | 105497 | 31824 |
| S/D Day 12 | 4.33 ± 0.22 | 0.97 ± 0.01 | 39735 | 44976 |
| S/D Day 21 | 3.67 ± 0.29 | 0.93 ± 0.03 | 36171 | 31715 |
| D/S Day 2 | 4.55 ± NA | 0.98 ± NA | 2837 | 33358 |
| D/S Day 6 | 4.44 ± 0.30 | 0.97 ± 0.01 | 60553 | 34248 |
| D/S Day 9 | 4.49 ± 0.13 | 0.98 ± 0.00 | 50753 | 39359 |
| D/S Day 12 | 4.43 ± 0.08 | 0.98 ± 0.00 | 37056 | 29113 |
| D/S Day 21 | 4.51 ± 0.10 | 0.98 ± 0.00 | 39678 | 42024 |

Note: Day 0 filters were lost

***
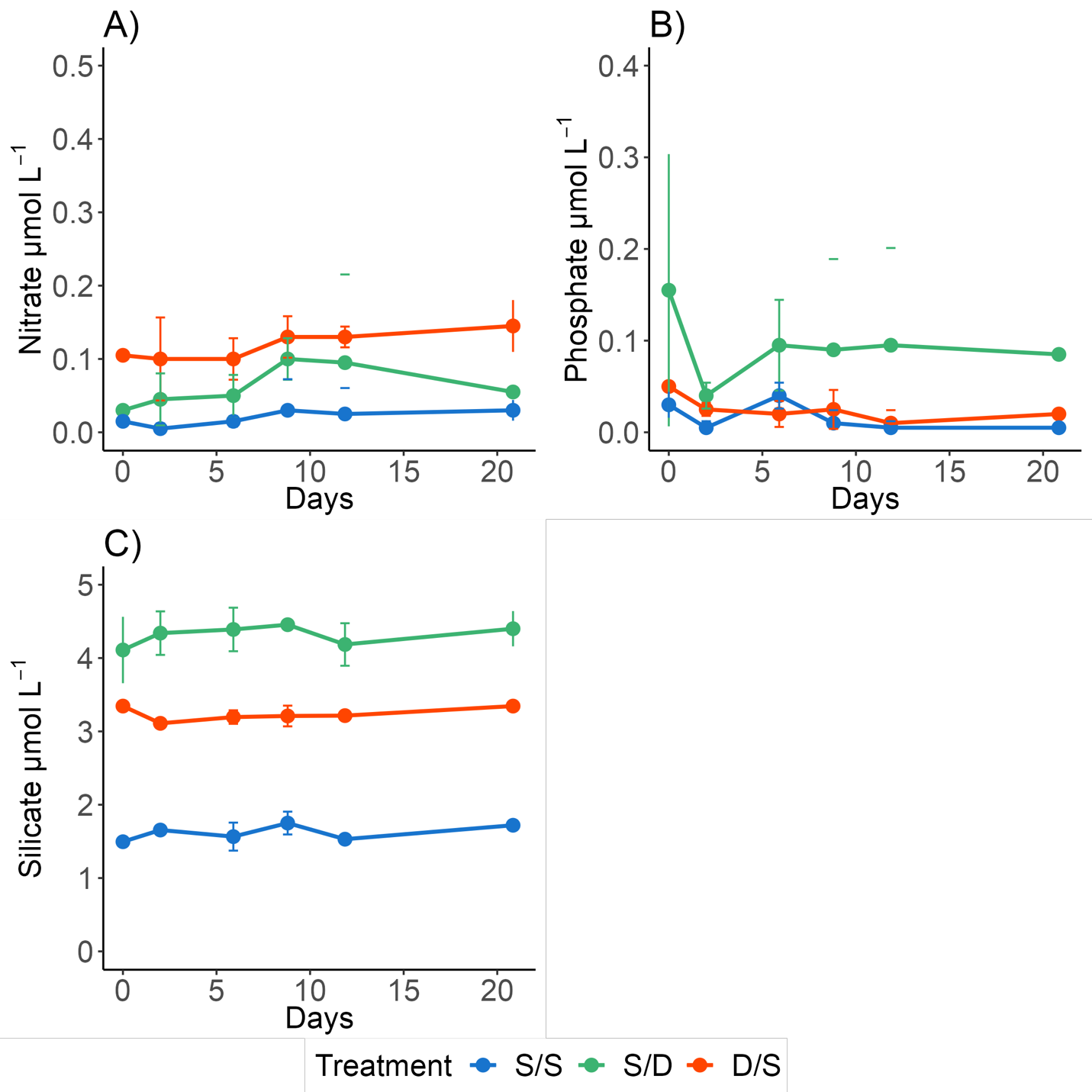
***

b

***Figure S1:*** *Line graphs of nutrients concentrations for A) Nitrate (µmol L^-1^); B) Phosphate (µmol L^-1^) and C) Silicate (µmol L^-1^) for all three treatments over 21 days.*

***
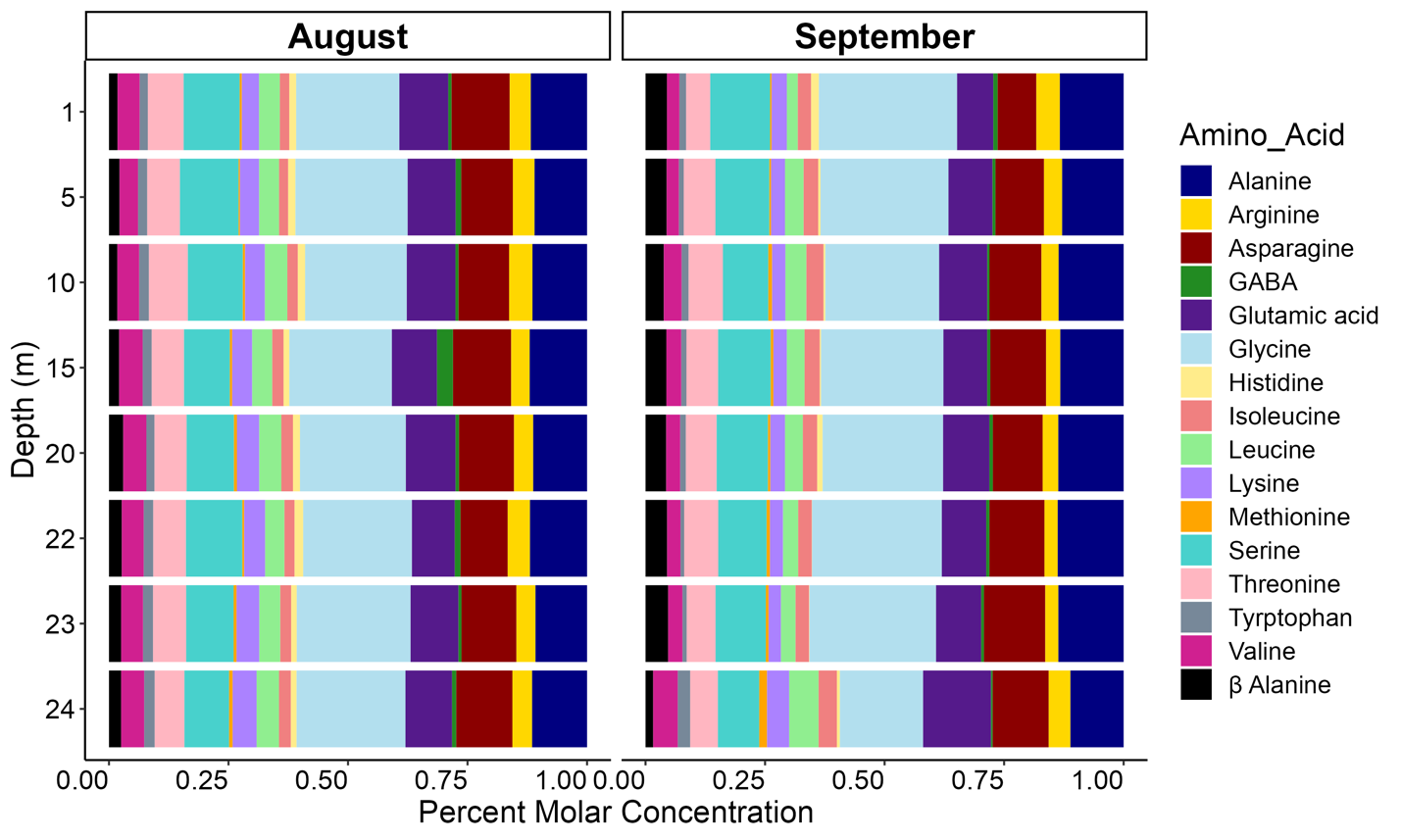
***

***Figure S2:*** *Stacked bar plots of the percent molar concentrations for the amino acid data for the depth profile taken at the time of the experimental set-up in August 2018 and just before convective overturn in September 2018.*

***
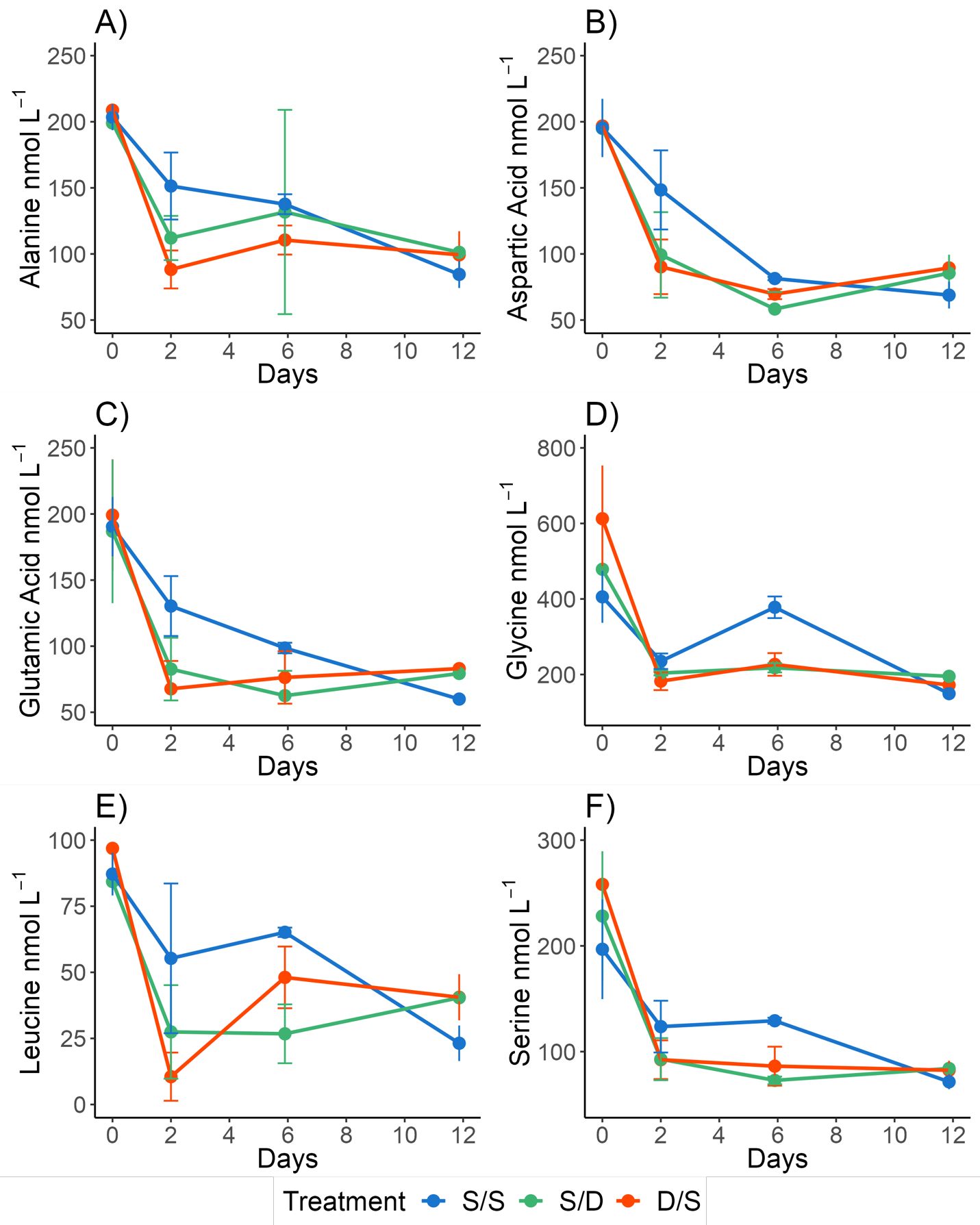
***

***Figure S3:*** *Line graphs of amino acid concentrations in nmol L^-1^for A) Alanine; B) Aspartic Acid; C) Glutamic Acid; D) Glycine and E) Leucine and F) Serine for all three treatments over 12 days.*
